# The obese inflammatory microenvironment may promote breast DCIS progression

**DOI:** 10.3389/fimmu.2024.1384354

**Published:** 2024-07-12

**Authors:** Ola Habanjar, Rawan Nehme, Nicolas Goncalves-Mendes, Gwendal Cueff, Christelle Blavignac, Jessy Aoun, Caroline Decombat, Céline Auxenfans, Mona Diab-Assaf, Florence Caldefie-Chézet, Laetitia Delort

**Affiliations:** ^1^ Université Clermont-Auvergne, INRAE, UNH, Clermont-Ferrand, France; ^2^ Université Clermont-Auvergne, Centre d’Imagerie Cellulaire Santé (CCIS), Clermont-Ferrand, France; ^3^ Banque de tissus et de cellules, Hôpital Edouard-Herriot, Lyon, France; ^4^ Equipe Tumorigénèse Moléculaire et Pharmacologie Anticancéreuse, Faculté des Sciences II, Université libanaise Fanar, Beirut, Lebanon

**Keywords:** ductal carcinoma *in situ*, obesity, microenvironment, inflammation, tumoroid, myoepithelial cells, macrophages

## Abstract

**Introduction:**

Ductal carcinoma *in situ* (DCIS), characterized by a proliferation of neoplastic cells confined within the mammary ducts, is distinctly isolated from the surrounding stroma by an almost uninterrupted layer of myoepithelial cells (MECs) and by the basement membrane. Heightened interactions within the adipose microenvironment, particularly in obese patients, may play a key role in the transition from DCIS to invasive ductal carcinoma (IDC), which is attracting growing interest in scientific research. Adipose tissue undergoes metabolic changes in obesity, impacting adipokine secretion and promoting chronic inflammation. This study aimed to assess the interactions between DCIS, including *in situ* cancer cells and MECs, and the various components of its inflammatory adipose microenvironment (adipocytes and macrophages).

**Methods:**

To this end, a 3D co-culture model was developed using bicellular bi-fluorescent DCIS-like tumoroids, adipose cells, and macrophages to investigate the influence of the inflammatory adipose microenvironment on DCIS progression.

**Results:**

The 3D co-culture model demonstrated an inhibition of the expression of genes involved in apoptosis (*BAX, BAG1, BCL2, CASP3, CASP8*, and *CASP9*), and an increase in genes related to cell survival (*TP53*, *JUN*, and *TGFB1*), inflammation (*TNF-α, PTGS2, IL-6R*), invasion and metastasis (TIMP1 and MMP-9) in cancer cells of the tumoroids under inflammatory conditions versus a non-inflammatory microenvironment. On the contrary, it confirmed the compromised functionality of MECs, resulting in the loss of their protective effects against cancer cells. Adipocytes from obese women showed a significant increase in the expression of all studied myofibroblast-associated genes (myoCAFs), such as *FAP* and *α-SMA*. In contrast, adipocytes from normal-weight women expressed markers of inflammatory fibroblast phenotypes (iCAF) characterized by a significant increase in the expression of *LIF* and inflammatory cytokines such as *TNF-α, IL-1β, IL-8*, and *CXCL-10*. These changes also influenced macrophage polarization, leading to a pro-inflammatory M1 phenotype. In contrast, myoCAF-associated adipocytes, and the cancer-promoting microenvironment polarized macrophages towards an M2 phenotype, characterized by high CD163 receptor expression and IL-10 and TGF-β secretion.

**Discussion:**

Reciprocal interactions between the tumoroid and its microenvironment, particularly in obesity, led to transcriptomic changes in adipocytes and macrophages, may participate in breast cancer progression while disrupting the integrity of the MEC layer. These results underlined the importance of adipose tissue in cancer progression.

## Highlights

- Development of a 3D co-culture model reproducing the inflammatory adipose tumor microenvironment of DCIS in obese patients.
- Investigation of the bidirectional communication between DCIS-like tumoroid and its microenvironment (adipose cells, macrophages).
- Exploring the impact of obesity-related chronic low-grade inflammation on DCIS development and progression.
- Repression of the expression of genes involved in apoptosis and stimulation of those involved in cell survival and inflammation in obesity.
- Compromised functionality of myoepithelial cells, leading to loss of their protective effects against cancer cells.
- Suppression of adipocyte-differentiation-related genes by DCIS-like tumoroid.
- Importance of understanding these interactions for unraveling tumor progression mechanisms and identifying potential therapeutic targets.

## 1 Introduction

Ductal carcinoma *in situ* (DCIS) is an intraductal neoplastic proliferation of atypical epithelium separated from the surrounding stroma by an almost continuous layer of myoepithelium and basement membrane (1). The term “*in situ*” means that cancer cells are confined within the ductal system and have not disseminated into adjacent tissues. Typically identified through mammography screenings, DCIS, despite being non-invasive, has been substantiated by various clinical observational studies as a potential precursor to invasive ductal mammary cancer (IDC) (2). If left untreated or inadequately managed, an estimated 30% of DCIS cases may progress, invading the contiguous breast tissue and evolving into IDC (3, 4).

As per the American Cancer Society’s 2021 estimates for the United States, around 49,290 new DCIS cases and 268,600 IDC cases were diagnosed in women, constituting approximately 20% to 25% of all breast cancers documented in the literature (5). Consequently, DCIS has emerged as a significant clinical challenge due to its escalating incidence and the potential for progression to IDC. The myoepithelial cell (MEC) layer, identifiable through specific markers such as p63 and α-smooth muscle actin (α-SMA) (6), plays a pivotal role in the normal breast structure. This layer acts as a “natural tumor suppressor,” regulating both normal mammary epithelial development and preventing cancer invasion, distinguishing DCIS from IDC (7).

Cancer cells derived from luminal epithelial cells establish direct contact with the stroma and adipose tissue before becoming invasive (8). Barsky and coworkers were the first to use functional assays to show that MECs have numerous antitumorigenic properties, such as the ability to inhibit tumor cell invasion and angiogenesis (9, 10). Research studies have demonstrated significant gene expression changes in MECs within the tumor microenvironment (TME) between DCIS and IDC. These alterations involve alterations in the expression of genes related to cell adhesion, extracellular matrix remodeling, invasion, and metastasis, particularly influencing leptin and inflammation-related genes (11). Notably, MECs are deemed critical in both the maintenance of DCIS and the invasion process (12). The involvement of the adipose microenvironment, particularly in obese patients, and the degradation of the basement membrane, are important factors in this process as well as an area of active investigation (13).

Mammary epithelia are surrounded by mammary adipose tissue, constituting the major component of the TME. It is composed of several cell types, including adipose stem cells (ASCs), preadipocytes (PA), mature adipocytes (MA), fibroblasts, and endothelial and immune cells, which have a range of functions (14). Numerous investigations demonstrate that stromal cells within the breast adipose tissue adjacent to the tumor establish a distinct TME capable of intricately modulating cancer progression (15). This regulatory capacity derives from the intricate bidirectional crosstalk among cancer cells, adipose cells, and immune cells (16). Adipocytes, originally considered as a simple energy storage depot, are now recognized as endocrine cells releasing a variety of hormones, growth factors, chemokines, and adipokines (17). The process of adipocyte differentiation begins with ASCs, which are multipotent cells found in various tissues that can differentiate into multiple cell types, including adipocytes. The committed ASCs undergo proliferation and differentiate into pre-adipocytes (PAs) which are considered intermediate cells possessing the potential to further differentiate into mature adipocytes (MAs) (18). PAs undergo growth arrest and begin to accumulate lipid droplets within the cytoplasm, which is accompanied by the upregulation of genes involved in lipid metabolism, such as adipocyte protein 2 (AP2) and the main adipogenic transcription factor, peroxisome proliferator-activated receptor gamma (PPAR-γ). The cells acquire the characteristic features of MAs, including a round shape, a large lipid-filled vacuole, and the ability to secrete adipokines such as adiponectin and leptin (19, 20).

The adipose tissue is an endocrine organ as well as an immune organ, as it is physiologically infiltrated by innate immune cells. Adipocytes and immune cells have a strong potential to influence tumor behavior and cancer aggressiveness through heterotypic signaling of soluble factors such as cytokines and growth factors (21). Adipose tissue can exert both paracrine and endocrine effects on breast tumor development. In the early stages of carcinogenesis and during breast cancer evasion, TME can be reorganized by cancer cells to generate a TME favorable for cancer cell proliferation and invasion into the surrounding tissue (22). In addition, recent work suggests that breast cancer cells may stimulate the dedifferentiation of MAs resulting in phenotypic changes and the generation of fibroblast-like cells *i.e.* adipocyte-derived fibroblasts (ADF) (23). In addition, TME plays an important role in macrophage polarization. Macrophages constitute a highly heterogeneous population of cells that undergo extensive changes in their intracellular metabolism in response to environmental and inflammatory stimuli. Unpolarized macrophages (M0) can differentiate into either classically activated pro-inflammatory macrophages (M1-like macrophages) or activated anti-inflammatory macrophages (M2-like macrophages M2a, M2b, M2c, M2d) or immunosuppressive tumor-associated macrophages (TAM) type M2, which play a central role as tumor-protecting cells, in breast cancer growth and progression (24). In lean animals, the adipose tissue microenvironment is predominantly composed of M2 macrophages, maintaining a ratio of approximately 4:1 compared to M1 macrophages. However, with the onset of obesity, this equilibrium is significantly disrupted, shifting to a ratio of roughly 1.2:1 in favor of M1 macrophages. This shift is attributed to the augmented recruitment and infiltration of pro-inflammatory M1 macrophages into the adipose tissue, which in turn exacerbates tissue inflammation (25–27).

Several studies have investigated the association between obesity and DCIS and have found a positive correlation. Obesity, classified according to a body mass index (BMI) ≥ 30 kg/m^2^ (28), is associated with an increased risk of breast cancer, particularly in postmenopausal women (29). In addition, obesity increases the risk of death in pre-and post-menopausal breast cancer patients, but the risk is lower in premenopausal than in postmenopausal women (depending on estrogen receptor status) (30–32). In obese subjects, adipose tissue expansion leads to biological dysfunction and the creation of chronic low-grade inflammation. Thus, the secretory and metabolic profiles of adipocytes are affected by increased secretion of pro-inflammatory adipokines that could promote breast cancer development, growth, and progression (33). Furthermore, studies have shown that by stimulating inflammatory pathways, there is a correlation between the degree of adiposity/obesity and adipocyte size and number (34, 35), chronic low-level inflammation with less secretion of anti-inflammatory adipokines (adiponectin) and higher secretions of pro-inflammatory proteins (TNF-α, Leptin, IL-6, IFN-γ, and TGF-β1) which may lead to an increased risk of cancer (36). Overweight and obesity can also stimulate macrophage recruitment and the repolarization of macrophages from the M2-like to the M1-like phenotype. So, reciprocal interactions between adipocytes, macrophages, and breast cancer cells drive functional effects on the behavior of these cells. The adipose tissue can act on MECs and may potentially contribute to the loss of their tumor suppressor status. Understanding this interplay between the adipose tissue and MECs within the DCIS TME represents an important challenge to better recognize the DCIS to IDC progression. By secreting various adipokines, cytokines, and growth factors, the adipose tissue can modulate the surrounding microenvironment, alter the MEC phenotype and viability (11), and affect the surrounding stromal cells, immune cells, or cancer cells, as a result influencing tumor progression and invasion (16).

Therefore, our work aimed to develop and optimize a 3D co-culture model to evaluate the reciprocal interactions between the TME (adipose cells and macrophages) and the DCIS (MECs and cancer cells) and to better understand the impact of this crosstalk on the possible progression to IDC. A co-culture system between i) bicellular fluorescent DCIS-like tumoroids previously developed constituted by DCIS cells (MCF-10DCIS-mcherry) surrounded by MECs (Hs578bst-GFP), and ii) adipose cells (PAs or MAs) organized into spheroids and iii) macrophages (M0 or M1-type) was realized to study the crosstalk and cell interactions that can occur indirectly *via* soluble factors and secretions released by all cell types. In this article, an obese inflammatory microenvironment is reproduced and compared to the microenvironment of a normal-weighted patient by examining cytokine-activated/suppressed signaling pathways, changes in the microenvironment, and effects of inflammation. Mounting evidence suggests that the breast TME, composed of cancer cells, MECs, immune cells, and adipose cells, is a key factor in promoting DCIS to IDC transition, particularly in obese patients.

## 2 Materials and methods

### 2.1 Cell culture

#### 2.1.1 Myoepithelial cells

Human Hs578Bst myoepithelial cells (RRID: CVCL_0807) (MECs) were obtained and certificated by the American Type Culture Collection (LGC standard, ATCC, HTB-125, Cheyenne, WY, Laramie County). They were transduced using EF1A-EGFP (Vector Builder ID: VB900083-7716 grp) (multiplicity of infection = 40) and then sorted to select cells exhibiting similar size and fluorescent signals according to the previously detailed protocol (6). Hs578Bst-GFP were expanded in a growth medium consisting of Dulbecco’s Modified Eagle Medium/Ham’s F-12 (DMEM/F-12, Gibco, ThermoFisher Scientific, Waltham, MA, USA), supplemented with 50 µg.mL^−1^ gentamycin (ThermoFisher Scientific), 10% fetal bovine serum (FBS; Eurobio Scientific, Saclay, France), 2 mM L-glutamine (L-Gln, Gibco), and 30 ng.mL^−1^ epidermal growth factor (EGF, Merck Millipore, Burlington, Massachusetts, USA) at 37°C and 5% CO_2_. The growth medium was changed 2–3 times per week. At 80–85% confluence, cells were passaged by using 0.25% w/v trypsin-EDTA 0.53 mM solution (ThermoFisher Scientific) and seeded with the recommended ratio 1:3. Cells were used for experiments before passage 22 to prevent the risk of senescence. All experiments were cultured in a 5% CO_2_-humidified incubator at 37°C.

#### 2.1.2 Cancer cells

The human breast cancer cell line MCF10DCIS (RRID: CVCL_5552) (DCIS) was obtained and certified by Wayne State University. They were transduced using EF1A-mCherry (Vector Builder ID: VB900084-0158zxv) (multiplicity of infection = 5) and then sorted to select cells exhibiting similar size and fluorescent signals according to the previously detailed protocol (6). MCF10-DCIS-mcherry was expanded in a growth medium consisting of DMEM/F-12 (Gibco), supplemented with 50 µg.mL^−1^ gentamycin, 5% horse serum (Gibco), 2 mM L-Gln, 1.1 mM CaCl_2_ (Merck Millipore) and 10 mM HEPES (Gibco) at 37°C and 5% CO_2_. The growth medium was replaced every 2 days. At 80–85% confluence, cells were passaged using 0.25% w/v trypsin-EDTA 0.53 mM solution. All experiments were cultured in a 5% CO_2_-humidified incubator at 37°C.

#### 2.1.3 Adipose cells

Human adipose stem cells (hASCs) were provided by the Cell and Tissue Bank (Hôpital Edouard-Herriot, Lyon, France). They were obtained from patients undergoing surgery for cosmetic purposes without associated pathology in accordance with the Helsinki Declaration, from anonymous healthy donors. The surgical residue was harvested following French regulations including a declaration of Ministry of Higher Education and Research of France (DC no.2008162) and procurement of written informed consent from the patients. hASCs were extracted from postmenopausal normal weight (Nw) or obese (Ob) postmenopausal women. The characterization of hASCs was performed as previously described (37).

hASCs were differentiated into PAs by culturing them in a differentiation medium validated by our team, consisting of consisting of DMEM/F-12, supplemented with 50 µg.mL^-1^ gentamycin, 10% FBS, 2 mM L-Gln, 5 μg.mL^-1^ basic-fibroblast growth factor (bFGF) (Sigma-Aldrich, St. Louis, MO, USA) at 37°C and 5% CO_2_ (37). The growth medium was replaced every 2 days. At 80-85% confluence, cells were passaged using 0.25% w/v trypsin-EDTA 0.53 mM solution. All experiments were cultured in a 5% CO_2_-humidified incubator at 37°C.

For differentiation into MAs, hASCs were seeded at the confluence of 33,500 cells/c in a differentiation medium validated by our team consisting of DMEM/F12 supplemented with 10% FBS, 2 mM L-Gln, hydrocortisone (25 mg.mL^-1^), insulin (3.5 mg.mL^-1^), T3 (6.5 mg.mL^-1^), dexamethasone (980 mg.mL^-1^), rosiglitazone (1.78 mg.mL^-1^), isobutyl-methylxanthine (IBMX) (100 mg.mL^-1^, only for the first 3 days), and gentamycin (50 mg.mL^-1^). The medium was replaced every two days (38). MAs of normal weight (Nw-MA) or obese (Ob-MA) women were obtained after 8 days of differentiation. All experiments were cultured in a 5% CO_2_-humidified incubator at 37°C. The evaluation of MA differentiation efficiency was carried out as previously detailed (37, 38).

#### 2.1.4 Macrophages

The human monocytic leukemia cell line (THP-1) (ATCC, TIB-202) was cultured in a growth medium consisting of RPMI-1640 medium supplemented with 50 µg. mL^-1^ gentamycin, 10% FBS and 2 mM L-Gln at 37°C and 5% CO_2_. The growth medium was replaced every 2-3 days. For the activation of THP-1 cells into M0 macrophages, THP-1 cells (4 × 10^5^/mL) were incubated in 6-well plates in a complete growth medium containing 16.2 nM phorbol 12-myristate 13-acetate (PMA, Sigma-Aldrich) for 72h. Then, M0 macrophages were polarized into pro-inflammatory M1-like macrophages by incubation with 20 ng/mL of IFN-γ (Gibco) and 10 pg. mL^-1^ of lipopolysaccharides (LPS, Sigma-Aldrich) for 24 h at 37°C and 5% CO_2_. All experiments were cultured in a 5% CO_2_-humidified incubator at 37°C. The validation of the efficacy of the M0/M1 polarization was checked by flow cytometry.

#### 2.1.5 Flow cytometry

For adjustment, two macrophage subtypes were used: M0 and M1-like macrophages. Cells were collected and adjusted to a density of 1 × 10^6^cells.mL^-1^ with DPBS (PBS; without Ca^2+^ and Mg^2+^, pH 7.4). Then, 1 mL of cell suspension was dispensed into each of the polystyrene flow cytometry tubes for unstained controls, Fluorescence Minus One (FMO) control, and fully stained samples. Functional markers diluted to 1/25 in PBS were used to identify macrophages subtypes: M0 using CD14-PE-VIO 770-Human (130-110-521, Miltenyi Biotec) and M1 using CD80-PE-Human-REA661 (130-123-253, Miltenyi Biotec). To accurately gating cell populations, isotype controls were used diluted to 1/50 in PBS: REA-PE VIO770 (130-113-440, Miltenyi Biotec) and REA-PE REA293 (130-113-438, Miltenyi Biotec). To adjust viability staining, killed cells (with ethanol) were used and stained with Viobility dye (Miltenyi Biotec) at the same dilution as recommended by the manufacturer. All antibodies and isotypes were titrated to determine the optimal concentration considering separation (by staining index), reduction of overflow spread, and detection range of the compensation beads. Cells were first stained for viability using Viobility dye 405/520(130-130-404) for 10 min at 4°C. Then, cells were stained with 4 μL of CD14-PE-VIO-770-Human (130-110-521) and 4 μL of CD80-PE-Human-REA661(130-123-253) in a total volume of ∼100 μL for 15 min at 4°C and 15 min at room temperature. After staining, cells were washed once with PBS (without Ca^2+^ and Mg^2+^), centrifuged at 300 g for 5 min at RT, resuspended in 500 µL of PBS, and then placed at 4°C for acquisition. Samples were acquired within 1h of storage. Flow cytometry was performed, after optimization of the panel, using a BD-LSRII flow cytometer (minimum 30,000 live cells counted) to check macrophage activation (M0%) and polarization (M1%). Instrument setup and performance tracking were performed daily using instrument-specific Cytometer Setup and Tracking (CS&T) beads (BD) using the CS&T program. Results were analyzed with FACSDiva version 9.1 software (BD Biosciences, Becton, Dickinson, and Company headquarters). Macrophages undergo active development and exhibit distinct polarization states, characterized by their response to inflammation. They were categorized into two main phenotypes: non-inflammatory M0 (90% CD14^+^&CD80^-^) and inflammatory M1 macrophages(90%CD80^+^). These macrophage subtypes can be effectively distinguished using specific antibodies that target unique markers on their cell surfaces (**Supplementary Materials No 1**).

### 2.2 Spheroid generation

The objective was to generate 3D spheroids using non-adherent agarose mold gel in which the cells cannot adhere to the support and thus can multiply in 3D and form multicellular micro-tissues. Agarose (ThermoFisher Scientific)was prepared with sterilized 0.9% w/v NaCl (Sigma-Aldrich), subsequently sterilized, and then put in MicroTissues^®^ 3D Petri Dishes^®^ (81 wells, Sigma-Aldrich) according to the manufacturer’s instructions to obtain an agarose mold (6, 39).

#### 2.2.1 Adipospheroid generation

Two types of spheroids have been developed: Pre-adipospheroids made up of PA and Adipospheroids made up of MAs.

For pre-adipospheroids, a total of 200,000 hASCs/agarose mold were seeded in the agarose mold and cultured in DMEM/F12 medium (supplemented with 10% FBS, 2 mM L-Gln, 50 µg.mL^-1^ gentamycin, 5 μg.mL^-1^ bFGF) for 3 days at 37°C in 5% CO_2_, resulting in the assembly of 81 potential Nw-PA and Ob-PA pre-adipospheroids per agarose mold. Concerning the formation of adipospheroids, a total of 200,000 hASCs/agarose mold were seeded in agarose mold and cultured in DMEM/F12 medium (supplemented with 10% FBS, 2 mM L-Gln, 50 µg.mL^-1^ gentamycin, 5 μg.mL^-1^ bFGF) at 37°C in 5% CO_2_. On day 2, the medium was replaced by the differentiation medium (as described previously) (38), resulting in the assembly of 81 potential Nw-MA and Ob-MA adipospheroids per agarose mold.

#### 2.2.2 Bi-fluorescent DCIS-like tumoroid generation

A total of 100,000 MCF10DCIS-mcherry cells were seeded into each agarose mold and cultured in MCF10DCIS medium for 72 hours at 37°C in 5% CO_2_, which led to the assembly of 81 potential MCF10DCIS tumoroids per agarose mold according to the previously detailed protocol (6). After 72 hours of incubation, a total of 30,000 Hs578Bst-GFP cells were added to each agarose mold containing the previously formed MCF10DCIS tumoroids, slowly shaken for 20 min at room temperature, then cultured in MECs medium for 12 hours at 37°C in 5% CO_2_ (6). The cell culture medium penetrates the tumoroids by diffusion.

### 2.3 Effect of conditioned media

Conditioned media (CM) were collected from the culture of Nw-PA, Ob-PA, Nw-MA, and Ob-MA (same patient) after differentiation of Nw-PA and Ob-PA respectively. CM was also obtained from the culture of M0 and M1-type macrophages. To minimize oxidation of the CM and ensure better long-term preservation for subsequent analysis, the oxygen was removed from CM ampoules and replaced with nitrogen. All CM (n=3) were harvested and centrifuged at 12,000 g for 15 mins to remove debris. The samples were then frozen at -80°C until use.

The impact of the different CMs on bi-fluorescent DCIS–like tumoroid viability was monitored. For that, DCIS-like tumoroids previously formed in agarose molds were transferred to the wells of a 96-well ultra-low-binding U-shaped-bottom plate (Corning, Somerville, MA, USA) (one single tumoroid in one well) and cultured in 200 μL of undiluted CM for 72 hours at 37°C in 5% CO_2_. Using IncuCyte^®^ (Sartorius, Göttingen, Allemagne), green and red fluorescence intensity variation was measured to monitor the cell viability of each cell line and monitor the impact of obesity, adipose, and inflammatory microenvironments on cancer cells and MECs viability (n=3 for each CM).

### 2.4 3D co-culture model

**Figure 1:**
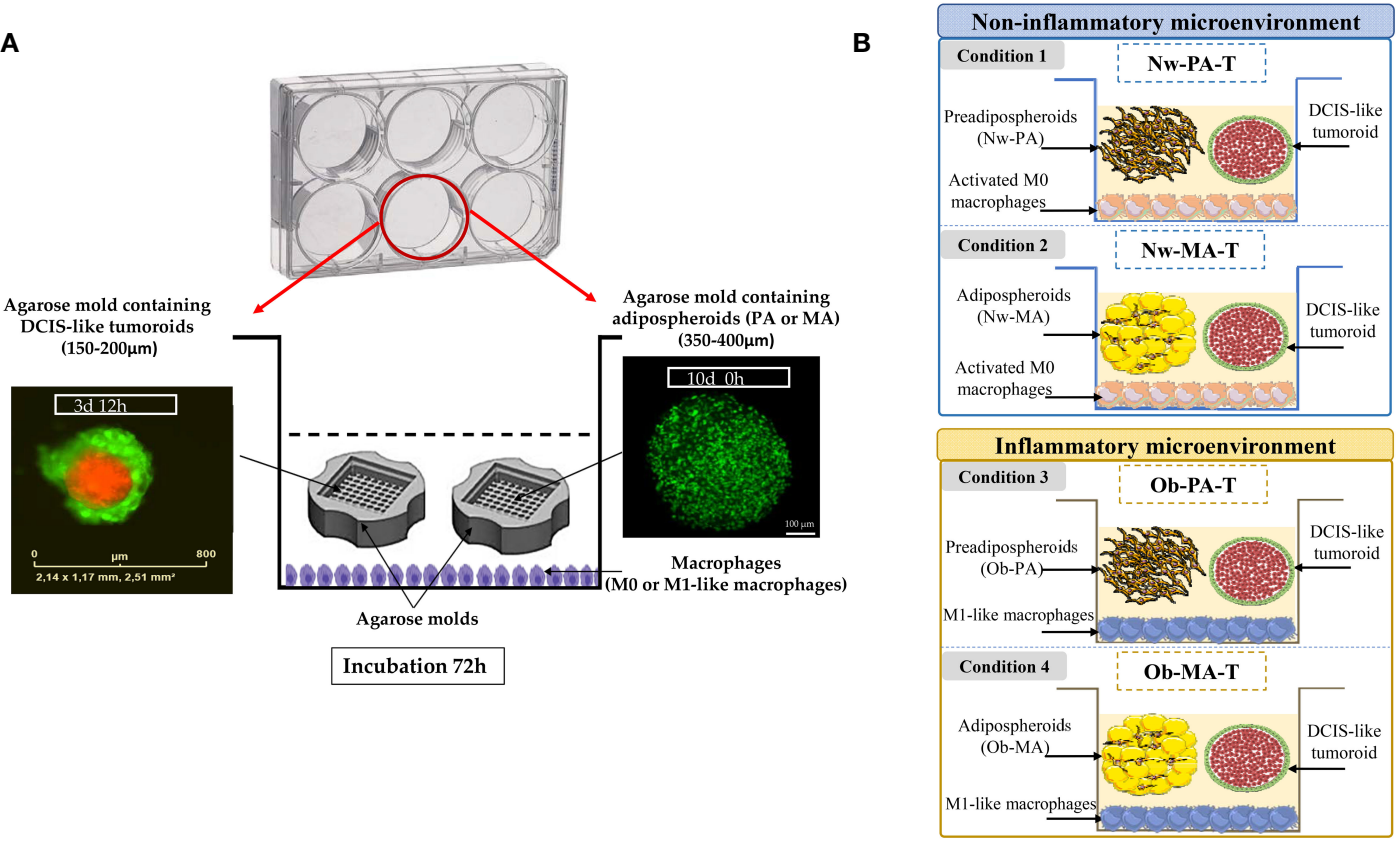
**(A)** Co-culture model mimicking a DCIS surrounding by its adipose inflammatory microenvironment. THP-1 cells (4 × 10^5^/mL) were incubated in 6-well plates in a complete growth medium for three days, differentiated in macrophages (M0), and polarized into M1-like macrophages by incubation with IFN-γ and lipopolysaccharides for 24 h. M0 or M1-like macrophages were co-incubated with two-cell-type, DCIS-like tumoroids (IncuCyte^®^ objective ×4) and adipospheroids (PA or MA) both formed in the agarose mold (confocal microscopy of adipospheroids stained with Bodipy, Micro Zeiss Cell Observer Spinning Disk, ×20 Plan Apochromat 20×/0.8 M27). **(B)** Description of the experimental design. A total of 4 conditions (3 independent experiments) mimicking a Nw- and Ob-TME were generated: Condition 1 *Nw-PA-T*: “Normal-weight tumor microenvironment with PA”, co-culture of M0 + NwPA + DCIS-like tumoroids, Condition 2 *Nw-MA-T*: “Normal-weight tumor microenvironment with MA”, co-culture of M0 + NwMA + DCIS-like tumoroids, Condition 3 *Ob-PA-T*: “Obese inflammatory adipose tissue with PA”, co-culture of M1-like macrophages + Ob-PA + DCIS-like tumoroids, Condition 4 *Ob-MA-T*: “Obese inflammatory adipose tumor microenvironment with MA”, co-culture of M1-like macrophages + Ob-MA + DCIS-like tumoroids.

A 3D co-culture system was set up to evaluate the reciprocal interactions between DCIS-like tumoroids and the adipose microenvironment (**Figure 1**) mimicking either an inflammatory adipose microenvironment found in obese people (Ob-TME), or a standard adipose microenvironment found in normal-weight individuals (Nw-TME).

#### 2.4.1 Model implementation

For the 3D model mimicking a Nw-TME, THP-1 monocytes were first seeded at the bottom of the wells of a 6-well plate and activated in M0 according to the protocol detailed above. Then these macrophages were co-cultured for 72h with both agarose molds containing DCIS-like tumoroids and adipospheroids made up with either Nw-PA or Nw-MA (Conditions 1 and 2) (**Figure 1B**) and compared to their respective controls without tumoroids (Control 1: Nw-PA-M0; Control 2: Nw-MA-M0) (**Supplementary Materials No 2**).

For the 3D model mimicking an obese inflammatory adipose microenvironment, THP-1 monocytes were polarized into M1-like pro-inflammatory macrophages, representing the predominant phenotype within the inflammatory microenvironment associated with obesity. Subsequently, these macrophages were co-cultured with both DCIS-like tumoroids and adipospheroids constituted by Ob-PA or Ob-MA (Conditions 3 and 4) (**Figure 1B**) and compared to their respective controls (Control 3: Ob-PA-M1; Control 4: Ob-MA-M1) (**Supplementary Materials No 2**).

Cells were co-cultured in a medium containing 7 mL of DMEM/F-12 supplemented with 50 µg. mL^-1^ gentamicin, 10% fetal bovine serum (FBS), 2 mM L-glutamine (L-Gln), and 30 ng. mL^-1^ epidermal growth factor (EGF). This medium represents the joint co-culture-conditioned media facilitating paracrine communications among distinct cellular types.

After 72 hours of incubation, the co-culture-conditioned medium was utilized for the quantification and identification of various cytokines across all co-culture conditions. The total RNA from each cell type was extracted using TRIZOL (ThermoFisher Scientific), and RT-qPCRs were subsequently conducted. These experiments were conducted independently at least three times.

A total of 2 conditions (3 independent experiments) mimicking an Nw-TME were generated (**Figure 1B**):

о Condition 1 *Nw-PA-T*: “Normal-weight tumor microenvironment with PA”, co-culture of M0 + NwPA + DCIS-like tumoroids *vs* Control 1 *Nw-PA-M0* “Normal-weight adipose tissue with PA”, co-culture of M0 + Nw-PA.
о Condition 2 *Nw-MA-T*: “Normal-weight tumor microenvironment with MA”, co-culture of M0 + NwMA + DCIS-like tumoroids *vs* Control 2 *Nw-MA-M0:* “Normal-weight adipose tissue with MA”, co-culture of M0 + Nw-MA.

A total of 2 conditions (3 independent experiments) mimicking an Ob-TME were generated (**Figure 1B**):

о Condition 3 *Ob-PA-T*: “Obese inflammatory adipose tissue with PA”, co-culture of M1-like macrophages + Ob-PA + DCIS-like tumoroids *vs* Control 3 *Ob-PA-M1*: “Obese inflammatory adipose tissue with PA”, co-culture of M1-like macrophages + Ob-PA.
о Condition 4 *Ob-MA-T*: “Obese inflammatory adipose tumor microenvironment with MA”, co-culture of M1-like macrophages + Ob-MA + DCIS-like tumoroids *vs* Control 4 *Ob-MA-M1*:” Obese inflammatory adipose tissue with MA”, co-culture of M1-like macrophages + Ob-MA.

#### 2.4.2 Selection and sorting

After co-culture, the bi-fluorescent DCIS-like tumoroids were trypsinized using 0.25% w/v trypsin-EDTA 0.53 mM solution (ThermoFisher Scientific) to obtain a cell suspension containing both cell types. A BD Cell Analyser FACSAria SORP Cell Analyzer/Sorter (BD Biosciences, Franklin Lakes, New Jersey, USA) was used to separate the two cell types according to fluorescent signal type and BD FACSDiva™ CS&T Research was used to monitor the performance of the cytometer each day and to generate reproducible data. The prerequisites necessary for this process were to adjust the selection using untransformed and non-fluorescent cells of each cell line, to know cell diameters, to choose an appropriate pressure to avoid cell shock, to filter the cells before passing them, and to eliminate aggregates (if any). Finally, cancer cells were recovered separately from the myoepithelial cells and total RNA was extracted with TRIZOL to perform qRT-PCR.

#### 2.4.3 Evaluation of gene expression by quantitative real-time PCR

Following the co-culture, total RNA was extracted with TRIZOL reagent (Invitrogen, ThermoFisher Scientific). After the evaluation of the quantity and purity of RNA (Tecan Spark^®^, Männedorf, Switzerland), DNase treatment was applied to remove any remaining genomic DNA (DNase I Amplification grade, Invitrogen) and cDNA reverse transcription (HighCap cDNA RT Kit RNAse inhib, Invitrogen) was performed according to the manufacturer’s recommendations. Concerning pre- and adipospheroids, macrophages, and MECs, amplification reaction assays were carried out using SYBRGreen PCR Master Mix (Applied Biosystems, Waltham, Massachusetts, USA) and primers (**Table 1**) on a StepOne™ machine (Applied Biosystems). The thermal cycling conditions were 50°C for 2 min followed by an initial denaturation step at 95°C for 10 min, 40 cycles at 95°C for 30 s, 60°C for 30 s, and 72°C for 30 s. The experiments were carried out in duplicate for each data point. The analysis was conducted on 7 genes for macrophages (*TNF-α, IL-6, IL-1β, IL-8, TGF-β, IL-10, CD163*), 13 genes for adipocytes (*PPAR-γ, AP2, HSL, Leptin, Adiponectin, TNF-α, IL-6, IL-1β, IL-8, CXCL-10, FAP, LIF, α-SMA*) and 4 genes for MECs (*BAX, PCNA, α-SMA, CDH1*). The reference genes *GAPDH* and *β-actin* were used as an internal control for the normalization of RNA quantity and quality differences among the samples.

**Table 1:** PCR primer sequences.

Gene	Species	Forward Primer sequence (5’-3’)	Reverse Primer sequence (5’-3’)
*GAPDH*	Human	CACATGGCCTCCAAGGAGTAA	TGAGGGTCTCTCTCTTCCTCTTGT
*β-actin*	Human	CCTGGCACCCAGCACAAT	GCCGATCCACACGGAGTACT
*Il-8*	Human	CTGGCCGTGGCTCTCTTG	CCTTGGCAAAACTGCACCTT
*Il-1β*	Human	CCTGTCCTGCGTGTTGAAAGA	GGGAACTGGGCAGACTCAAA
*Il-6*	Human	GCTGCAGGCACAGAACCA	ACTCCTTAAAGCTGCGCAGAA
*TNF-α*	Human	TCTTCTCGAACCCCGAGTGA	GGAGCTGCCCCTCAGCTT
*CXCL-10*	Human	GGAAATCGTGCGTGACATTA	AGGAAGGAAGGCTGGAAGAG
*PPAR-γ*	Human	GGATTCAGCTGGTCGATATCAC	GTTTCAGAAATGCCTTGCAGT
*Ap2*	Human	ATCACATCCCCATTCACACT	ACTTGTCTCCAGTGAAAACTTTG
*HSL*	Human	GCCTGGGCTTCCAGTTCAC	CCTGTCTCGTTGCGTTTGTAGT
*Leptin*	Human	CGGAGAGTACAGTGAGCCAAGA	CGGAATCTCGCTCTGTCATCA
*Adiponectin*	Human	CCCAAAGAGGAGAGGAA	TCAGAAACAGGACACAAC
*BAX*	Human	CCTGTGCACCAAGGTGCCGGAACT	CCACCCTGGTCTTGGATCCAGCCC
*CD163 *	Human	CGGTCTCTGTGATTTGTAACCAG	TACTATGCTTTCCCCATCCATC
*TGF-β*	Human	GACATCAAAAGATAACCACTC	TCTATGACAAGTTCAAGCAGA
*IL-10*	Human	GGGGGTTGAGGTATCAGAGGTAA	GCTCCAAGAGAAAGGCATCTACA
*LIF*	Human	GAAAGCTTTGGTAGGTTCTTCGTT	TGCAGGTCCAGCCATCAGA
*FAP*	Human	TCCAGTCTCCAGCTGGGAAT	GTTGGGAGACCCATGAATCTCT
*α-SMA*	Human	TGCCTGATGGGCAAGTGAT	TCTCTGGGCAGCGGAAAC
*PCNA*	Human	AGGCACTCAAGGACCTCATCA	GAGTCCATGCTCTGCAGGTTT
*CDH1*	Human	ACAGCCCCGCCTTATGATT	TCGGAACCGCTTCCTTCA

Concerning cancer cells, qPCRs were performed on plates designed by Applied Biosystems (TaqMan Array 96 well Fast Plate, Customformat 32, Applied Biosystems) using PowerUp SYBRgreen (Applied Biosystems) with TaqMAN on a Quantstudio 3 machine (Thermo Fisher). The thermal cycling conditions were 50°C for 2 min followed by an initial denaturation step at 95°C for 2 min, 40 cycles at 95°C for 1 s and 60°C for 20 s. The analysis was conducted on 20 genes (*ESR1, CYP19A1, ERBB2, PGR, TP53, JUN, TGFB1, BAX, BAG1, BCL2, CASP3, CASP8, CASP9, THBS1, TIMP1, VEGFa, MMP9, TNF-α, PTGS2, IL-6R*) and 3 references genes (*18S, GAPDH, HPRT1).* The reference genes *GAPDH* and *HPRT1* were used as an internal control for the normalization of RNA quantity and quality differences among the samples.

Genes were considered significantly expressed and their transcript was measurable if their corresponding Ct value was less than 35. The relative quantification method (RQ = 2^–ΔΔCT^) was used to calculate the relative gene expression of given samples with ΔΔCT = [ΔCT (sample1) − ΔCT (sample2)] and ΔCT = [CT (target gene) − CT (reference gene). Three independent experiments were performed. *p*<0.05 was considered significant.

Concerning the analysis of genes expressed in MECs, adipocytes, and macrophages, relative mRNA gene expression (RQ) was normalized to *GAPDH* and *β-actin* in the four conditions Nw-PA-T (M0+Nw-PA+Tumoroids), Nw-MA-T (M0+Nw-MA+Tumoroids), Ob-PA-T (M1+Ob-PA+Tumoroids), and Ob-MA-T (M1+Ob-MA +Tumoroids) and compared to their respective control 1, 2, 3 and 4 to evaluate the impact of DCIS-like tumoroids on each group. *The P-value* corresponded to the comparison between the control and condition.

Concerning the analysis of genes expressed in cancer cells, relative mRNA expression (RQ) of genes was normalized to *GAPDH* and *HPRT1* in the four conditions. The *p-value* corresponded to the comparison between co-cultured tumoroids compared to “Tumoroids alone”; the *p2-value* corresponded to the comparison between Nw-PA-T and Ob-PA-T; the *p3-value* corresponded to the comparison between Nw-MA-T and Ob-MA-T.

#### 2.4.4 Determination of cytokine concentrations

ProcartaPlex™ Immunoassays (ThermoFisher Scientific) was used for all assays. All samples were run in triplicate and were assayed for 12 human cytokines (IFN-γ, IL-12 p70, IL-1β, IL-2, IL-23, IL-6, IL-8, IL-17a, MIP-1α, leptin, adiponectin and TNF-α). Cytokine levels were measured using optimal concentrations of standards and antibodies in accordance with the manufacturer’s instructions. After completion of all the steps in the assay, the plates were read in the Luminex Bio-Plex 200 System (Biorad, France) and the data were analyzed using Bio-Plex Manager™ 4.1 software with five-parameter logistic regression (5PL) curve fitting.

#### 2.4.5 Statistical analyses

Most of the statistical analysis was done thanks to the R software with the RStudio IDE (R 4.2.2, RStudio 2022.07.2). All the plots created with R come from ggplot in its tidyverse implementation (1.3.2). At each step the data were prepared thanks to dplyr functions (1.0.10).

In qPCR analyses, the RQ (Relative Quantification) data have been analyzed. Firstly, heatmap was created through the pheatmap package (1.0.12) on the basis of group means of fully scaled values. In heatmap function, the “ward.D2” algorithm was chosen and an optimal number of clusters for both groups and genes was visually set. To better isolate clusters of genes, correlograms were drawn thanks to the corrplot package (0.92), based on parametric Pearson correlations coefficients run with the same ward.D2 agglomerative method. Finally, differential analysis on co-cultures has been performed on a series of genes. This was done with the tools of stats and lsmeans packages in the aforementioned versions. For cell viability statistical significance among several groups was assessed using one-way ANOVA followed by Tukey’s multiple comparisons test in GraphPad Prism software version 8 (GraphPad Software, San Diego, USA).

For flow cytom*etry data, relevant tools have been applied for these frequencies data, i.e. mainly the rcompanion package (2.4.21) and its methods. To evaluate the variations in cell type frequencies between co-culture, the Cochran-Mantel-Haenszel method has been chosen in a global and in a pairwise approach. To represent these variations, a barplot where all the co-culture has been normalized to a value of 1, helps compare the proportions of each labeling.

For cytokine concentration data, two-way ANOVA has been first performed to assess the variations of the twelve measured cytokines. This analysis considered the type of macrophage or adipocyte cells in the microenvironment as two predictors in linear models. Here the anova_test function from the rstatix package (0.7.1) was used in a type 2 setting to calculate *p-values* for main effects and interactions. In these linear models, contributions (effect size) were calculated thanks to the lsr package (0.5.2). To complete these results, a deeper analysis was conducted with the lsmeans package (2.30-0). With this tool, all pairwise comparisons are assessed for main and simple effects, giving the relevant *p-values* translated into CLD (Compact Letter Display) that help interpretations. For a one-way ANOVA style analysis, the replicates “group” factor has been treated with seventeen levels, each corresponding to an original combination of cell types. The basic stats package (4.2.2) was used with its a function to detect overall variations amongst these seventeen groups, with an fdr adjustment applied to *p-values*. On the same but standardized data, PCAs were produced to visualize each sample and group on a multivariate basis, the distributions being calculated from the twelve cytokines abundances. At this step, we used the FactoMineR (2.7) and factoextra (1.0.7) tools to generate PCAs.

## 3 Results

### 3.1 Impact of the adipose inflammatory microenvironment on DCIS-like tumoroids

#### 3.1.1 Impact of the adipose inflammatory microenvironment on cancer cells from DCIS-like tumoroids

**Figure 2:**
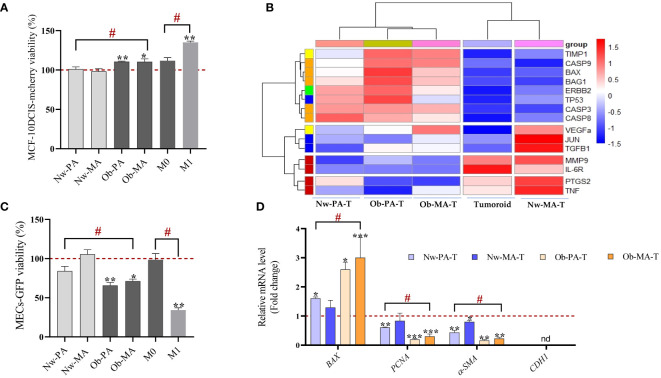
Impact of the adipose inflammatory microenvironment on cancer cells from DCIS-like tumoroids. **(A, B)** Impact of the adipose inflammatory microenvironment on cancer cells sorted from DCIS-like tumoroids. **(A)** Conditioned media from PA (Nw-PA, Ob-PA), MA (Nw-MA, Ob-MA) and macrophages (M0, M1) were obtained after 48h of culture and their impact on cell viability was assessed by measuring the fluorescence intensity using the IncuCyte^®^ system to monitor the fluorescence intensity of MCF10-DCIS-mcherry breast cancer cells. **(B)** Hierarchical clustering (heatmap) illustrates the variations of ΔCt mRNA expression of 15 genes normalized to GAPDH expressed by MCF10-DCIS cancer cells sorted from DCIS-like tumoroids co-cultured in the Nw-TME or the Ob-TME and compared to control (DCIS cells sorted from DCIS-like tumoroids control). The heatmap color code is categorized by gene type as follows: green for hormone-related genes, yellow for angiogenesis, blue for cell cycle, pro-oncogenes/transcription factors, red for inflammation, orange for apoptosis, and purple for proliferation. **(C, D)** Impact of the adipose inflammatory microenvironment on MECs sorted from DCIS-like tumoroids. **(C)** Conditioned media from PA (Nw-PA, Ob-PA), MA (Nw-MA, Ob-MA) and macrophages (M0, M1) were obtained after 48h of culture and their impact on cell viability was assessed by measuring the fluorescence intensity using the IncuCyte^®^ system to monitor the fluorescence intensity of Hs578Bst-GFP myoepithelial cells, M0: activated macrophages; M1: pro-inflammatory macrophages. **(D)** Relative mRNA expression (RQ) of 4 genes normalized to GAPDH expressed by MECs sorted from DCIS-like tumoroids co-cultured in the Nw-TME or the Ob-TME and compared to control (MECs sorted from DCIS-like tumoroids control). Results are expressed as percentage compared to each cultured media control (PA, MA, and macrophages cultured media). All data represent the means of 3-6 replicates ± SEM. * *p*<0.05, **p ≤ 0.01 ***p ≤ 0.001 represent significant differences compared to control and # represents a significant difference between normal-weight and obese condition. Nw-PA, adipose stem cells from normal-weight women; Ob-PA, adipose stem cells from obese women; Nw-MA, mature adipocytes from normal-weight women; Ob-MA, mature adipocytes from obese women; M0, activated macrophages; M1, pro-inflammatory macrophages. PCNA, Proliferating cell nuclear antigen; SMA-α, smooth muscle actin; CDH1, E-cadherin.

We first sought to identify the impact of the adipose inflammatory microenvironment on MCF-10DCIS-RFP cancer cells from the DCIS-like tumoroids by measuring the real-time viability by Incucyte^®^ and we showed that CM of Ob-PA, Ob-MA, and M1 significantly increased the percentage of red intensity which is proportional to the viability of MCF-10DCIS-RFP cancer cells (**Figure 2A**).

Then, we wanted to identify the underlying mechanism by which adipose tissue might act on cancer cells of DCIS-like tumoroids by focusing on changes in gene expression. We studied the modification of the expression of genes involved in apoptosis (*BAX, BAG1, BCL2, CASP3, CASP8, CASP9*), angiogenesis (*THBS1, TIMP1, VEGFα*), inflammation (*MMP9, TNF-α, PTGS2, IL-6R*), cell cycle/pro-oncogenesis/transcription factors (*TP53, JUN, TGFB1*), and cytokines/hormonal pathways (*ESR1, CYP19A1, ERBB2, PGR*).

Hierarchical clustering enabled clear discrimination between control tumoroids and tumoroids co-cultured with Nw-TME or Ob-TME with under-expressed genes (red font) and over-expressed genes (blue font) (**Figure 2B**). The heatmap revealed that the conditions Nw-PA-T, Ob-PA-T, and Ob-MA-T were particularly associated with an overexpression of genes related to inflammation and on the contrary with a down-regulation of genes related to apoptosis and angiogenesis. Nw-MA-T was associated with a lower expression of all genes related to cycle/pro-oncogene/transcription factor, VEGF, and TNF*-α*.

Apoptosis and cell cycle. All apoptosis-related genes (*BAX, BAG1, CASP3, CASP8*, and *CASP9* except *BCL2*), *TP53*, and *TGF-β* involved in cell growth were significantly expressed by cancer cells in all groups (**Tables 2**, **3**). The Ob-PA-T mainly decreased the expression of *BAX* (RQ=0.084; *p ≤* 0.001, *p2 ≤* 0.001), *BAG1* (RQ=0.101; *p ≤* 0.001, *p2 ≤* 0.05), and *TP53* (RQ=0.019, *p ≤* 0.001, *p2 ≤* 0.05) to a greater extent than Nw-PA-T (**Table 2**). In addition, Ob-MA-T mainly decreased the expression of all apoptosis-related genes such as *BAX* (RQ=0.21; *p ≤* 0.001, *p3 ≤* 0.001), *BAG1* (RQ=0.208; *p ≤* 0.001, *p3 ≤* 0.001), *CASP3* (RQ=0.215; *p ≤* 0.001, *p3 ≤* 0.001), *CASP8* (RQ=0.262; *p ≤* 0.001, *p3 ≤* 0.05), and *CASP9* (RQ=0.169; *p ≤* 0.001, *p3 ≤* 0.05) (**Table 3**). On the contrary, the expressions of *CASP3*, *CASP8*, and *CASP9* were similarly reduced in Ob-PA-T and Nw-PA-T. Nw-MA-T only slightly decreased the expression of *BAX* (RQ=0.8; p ≤ 0.01) and *CASP8* (RQ=0.551, p ≤ 0.05). The expression of *TP53* and *TGF-*β was reduced in Nw-MA-T and mainly in Ob-MA-T (**Table 3**).

**Table 2:** Relative mRNA expression (RQ) of 20 normalized genes expressed by tumor cells sorted from tumoroids co-cultured either with Nw—PA-T or Ob-PA-T and compared to Tumoroid control (*p-value*).

Category	Groups	Tumoroids	*Nw-PA-T*		*Ob-PA-T*		
	Genes	RQ	RQ	*p-value*	RQ	*p-value*	*p2-value*
**Apoptosis**	*BAX*	1	0.27	≤ 0.001	0.084	≤ 0.001	≤ 0.001
	*BAG1*	1	0.28	≤ 0.001	0.101	≤ 0.001	< 0.05
	*BCL2*	nd	nd	nd	nd	nd	nd
	*CASP3*	1	0.19	≤ 0.001	0.162	≤ 0.001	0.965
	*CASP8*	1	0.118	≤ 0.001	0.173	≤ 0.001	0.623
	*CASP9*	1	0.41	≤ 0.01	0.15	≤ 0.01	0.1723
**Angiogenesis**	*VEGFa*	1	0.522	≤ 0.01	0.43	≤ 0.01	0.391
	*THBS2*	nd	nd	nd	nd	nd	nd
	*TIMP1*	1	0.322	≤ 0.001	0.099	≤ 0.001	0.01
**Inflammation**	*MMP9*	1	40.861	≤ 0.001	10.456	≤ 0.001	≤ 0.001
	*PTGS2*	1	1.119	0.981	3.888	≤ 0.01	< 0.05
	*IL-6R*	1	2.54	< 0.05	2.132	< 0.05	0.998
	*TNF-α*	1	3	≤ 0.01	9.96	≤ 0.01	≤ 0.05
**Cycle/** **pro-oncogene/** **Transcription factor**	*TP53*	1	0.049	≤ 0.001	0.019	≤ 0.001	< 0.05
	*JUN*	1	0.899	0.587	1.067	0.7073	0.2340
	*TGFB1*	1	1.065	0.4677	0.466	≤ 0.001	≤ 0.001
**Hormonal pathway**	*ERBB2*	1	0.064	≤ 0.001	0.039	≤ 0.001	< 0.05
	*ESR1*	nd	nd	nd	nd	nd	nd
	*CYP19A1*	nd	nd	nd	nd	nd	nd
	*PGR*	nd	nd	nd	nd	nd	nd

*p2-value* corresponded to the comparison between Nw-PA-T and Ob-PA-T. nd, not determined.

**Table 3:** Relative mRNA expression (RQ) of 20 normalized genes expressed by tumor cells sorted from tumoroids co-cultured with Nw-MA-T or Ob-MA-T (*p-value*).

Category	Groups	Tumoroids	*Nw-MA-T*		*Ob-MA-T*		
	Genes	RQ	RQ	*p-value*	RQ	*p-value*	*p3-value*
**Apoptosis**	*BAX*	1	0.82	≤ 0.01	0.21	≤ 0.001	≤ 0.001
	*BAG1*	1	1.096	0.473	0.208	≤ 0.001	≤ 0.001
	*BCL2*	nd	nd	nd	nd	nd	nd
	*CASP3*	1	0.905	0.579	0.215	≤ 0.001	≤ 0.001
	*CASP8*	1	0.551	≤ 0.01	0.262	≤ 0.001	< 0.05
	*CASP9*	1	1.815	0.21	0.169	≤ 0.001	< 0.05
**Angiogenesis**	*VEGF a*	1	0.272	≤ 0.001	0.286	≤ 0.001	0.985
	*THBS2*	nd	nd	nd	nd	nd	nd
	*TIMP1*	1	0.523	≤ 0.001	0.121	≤ 0.001	≤ 0.001
**Inflammation**	*MMP9*	1	0.587	0.999	28.724	≤ 0.001	≤ 0.001
	*PTGS2*	1	0.454	0.245	4.163	≤ 0.001	≤ 0.001
	*IL-6R*	1	1.437	0.536	2.24	< 0.05	0.179
	*TNF-α*	1	0.20	0.0823	2	< 0.05	< 0.05
**Cycle/pro-oncogene/Transcription factor**	*TP53*	1	0.328	≤ 0.001	0.2	≤ 0.001	0.116
	*JUN*	1	0.211	< 0.05	0.784	0.555	0.0629
	*TGFB1*	1	0.173	≤ 0.001	0.546	< 0.05	≤ 0.001
**Hormonal pathway**	*ERBB2*	1	0.436	≤ 0.001	0.127	≤ 0.001	≤ 0.01
	*ESR1*	nd	nd	nd	nd	nd	nd
	*CYP19A1*	nd	nd	nd	nd	nd	nd
	*PGR*	nd	nd	nd	nd	nd	nd

*p3-value* corresponded to the comparison between Nw-MA-T and Ob-MA-T. nd, not determined.

Inflammation. All the studied genes involved in inflammation (*MMP9, PTGS2, TNF-α*, and *IL-6R*) were expressed by cancer cells. Ob-PA-T increased the expression of *PTGS2* and *TNF-α* expression by a factor of nearly 4 (RQ=3.888, *p ≤* 0.01, *p2 ≤* 0.05) and 10 (RQ=9.96, *p ≤* 0.001, *p2 ≤* 0.01) respectively (**Table 2**). In addition, Ob-MA-T had the most potent effect on the upregulation of all inflammatory genes by a factor near 28 for *MMP9* (RQ=28.724, *p ≤* 0.001, *p3 ≤* 0.001), 4 for *PTGS2* (RQ=4.163, *p ≤* 0.001, *p3 ≤* 0.001), 2 for *TNF-α* (RQ=2, *p*<0.05, *p3*<0.05) and for *IL-6R* (RQ=2.24, *p ≤* 0.05) which may be due to obesity-associated inflammation (**Table 3**). *IL-6R* expression was increased by a factor of 2 similarly within the Nw and Ob-PA-T which can be due to high IL-6 level in supernatant. *MMP9* expression increased by a factor of 40 within Nw-PA-T (RQ=40.861, *p ≤* 0.001), which is much higher than in Ob-PA-T in which expression increased 10-fold (RQ=10.456, *p ≤* 0.001, *p2 ≤* 0.001) (**Table 2**) while Nw-MA-T had a non-significant effect on all the inflammation-related gene expression.

Angiogenesis. A significantly decreased *TIMP1* expression in cancer cells was observed within Nw-PA-T (RQ=0.322, *p ≤* 0.001), Ob-PA-T (RQ=0.099, *p ≤* 0.001, *p2 =* 0.01) (**Table 2**), Nw-MA-T (RQ=0.579, *p ≤* 0.001) and Ob-MA-T (RQ=0.121, *p ≤* 0.001, *p3 ≤* 0.001) (**Table 3**), which can contribute to tumor migration and progression. Surprisingly, the expression of *VEGF* was similarly decreased in cancer cells within all TME groups.

Hormonal pathway. The hormonal-related genes studied (*ESR1, CYP19A1*, and *PGR*) were not expressed by MCF10-DCIS cancer cells that are HER2-enriched (ER−, PR−, HER2+/ERBB2+) intrinsic molecular subtypes of breast cancer (33). *ERBB2* (HER2) expression decreased in cancer cells co-cultured with Nw-PA-T (RQ=0.064, *p ≤* 0.001) (**Table 2**) and Nw-MA-T (RQ=0.436, *p ≤* 0.001) (**Table 3**), this expression continued to decrease in Ob-PA-T (RQ=0.039, *p ≤* 0.001, *p2*<0.05) (**Table 2**) and Ob-MA-T (RQ=0.127, *p ≤* 0.001, *p3*<0.01) (**Table 3**).

#### 3.1.2 Impact of the adipose inflammatory microenvironment on MECs sorted from DCIS-like tumoroids

As previously, we evaluated the impact of the adipose microenvironment on Hs578Bst-GFP MECs of the DCIS-like tumoroids, by measuring the real-time viability by Incucyte^®^. We found that only the CMs obtained from the inflammatory adipose microenvironment, *ie* Ob-PA, Ob-MA, and pro-inflammatory macrophages M1-like, significantly decreased the percentage of the green intensity that is proportional to the viability of the continuous layer of Hs578Bst-GFP MECs (**Figure 2C**). Then we studied the alteration of the expression of genes involved in apoptosis (*BAX*), DNA replication and repair processes (*PCNA*), and myoepithelial cell differentiation markers (*α-SMA*, *E-cadherin*) and all were significantly expressed by MECs in all conditions except *E-cadherin (*not expressed in the control and all experimental condition) (**Figure 2D**). We found that MECs co-cultured with the inflammatory adipose microenvironment presented the highest increase in *BAX* expression and the lowest decrease in *PCNA* and *α-SMA* expression. Indeed, the Ob-PA-T had a more potent effect than the Nw-PA-T on the downregulation of *PCNA* (RQ=0.192, *p ≤* 0.001) and *α-SMA* (RQ=0.165; *p ≤* 0.001) and the upregulation of *BAX* (RQ=2.5; *p ≤* 0.001). When we focused on the TME constituted with MA and M1-type macrophages, we observed that Ob-MA-T had a more potent effect than Nw-MA-T on *α-SMA* downregulation (RQ=0.22, *p ≤* 0.01). Ob-MA-T also increased *BAX* expression by a factor of 3 (RQ=3; *p ≤* 0.001) and decreased *PCNA* expression (RQ=0.3; *p ≤* 0.05) while Nw-MA-T did not affect the proliferation and pro-apoptotic activity of MECs of DCIS-like tumoroids but can affect its contractile properties.

### 3.2 Impact of DCIS-like tumoroids on its adipose inflammatory microenvironment

We investigated the underlying mechanisms by which DCIS-like tumoroids might act on adipose tissue by focusing on gene expression changes. We first measured the expression of genes involved in adipocyte differentiation (*PPAR-γ, AP2, HSL, Adiponectin, Leptin*), cancer-associated fibroblast (CAF) markers (*LIF, FAP, α-SMA*), and inflammation (*IL-6, IL-8, IL-1β, CXCL-10, TNF-α*). CAFs can exhibit diverse phenotypes and functions, influenced by various factors including tumor type, stage, and microenvironmental cues like cancer-associated myofibroblasts (myoCAF) characterized by the expression of α-SMA and FAP, and inflammatory cancer-associated fibroblast (iCAF) phenotypes characterized by expression of LIF and strong expression of inflammatory cytokines and chemokines. Then we evaluated the expression of genes involved in the inflammatory response and macrophage polarization (*IL-1β, IL-6, IL-8, TNF-α, TGF-β, IL-10, CD163*) in macrophages.

#### 3.2.1 The reprogramming of adipose cells within the TME

##### 3.2.1.1 DCIS-like tumoroids suppressed adipocyte-differentiation-related genes and affected adipokine secretions

**Figure 3:**
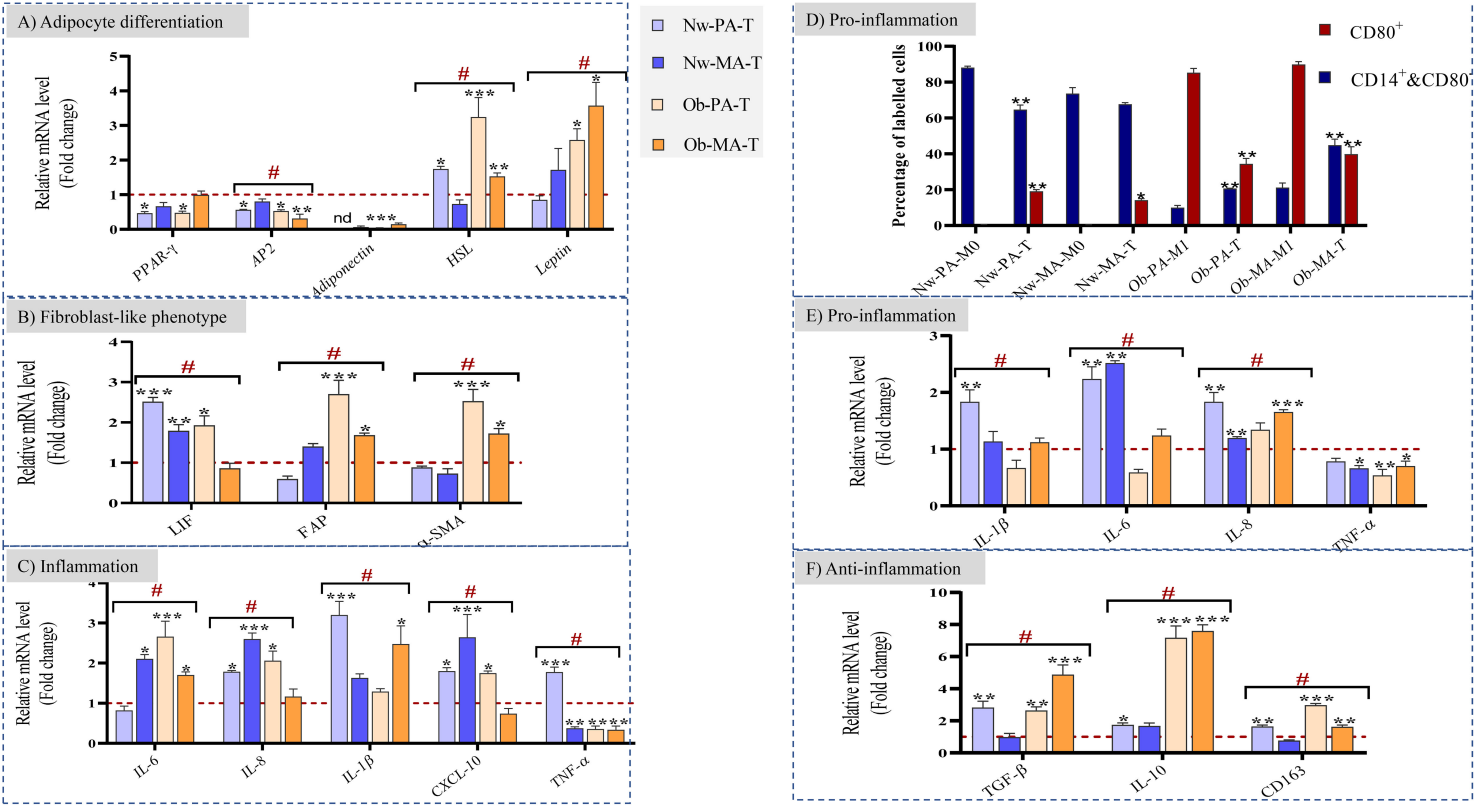
**(A–C)** Impact of the Nw-TME and Ob-TME on adipocyte differentiation, fibroblast-like phenotype and inflammation. Pre-adipospheroids (Nw-PA, Ob-PA) and adipospheroids (Nw-MA, Ob-MA) were co-cultured with macrophages (M0 or M1) and DCIS-like tumoroids. The mRNA relative expression of **(A)** adipocyte differentiation-related genes (*PPAR-γ, AP2, Adiponectin, HSL, Leptin*), **(B)** genes related to cancer-associated fibroblast phenotype (*LIF, FAP, α-SMA*) **(C)** genes related to inflammation (*IL-6, IL-8, IL-1β, CXCL-10, TNF-α*), and was measured by qRT-PCR. GAPDH was used as control. The four conditions Nw-PA-T (M0+Nw-PA+Tumoroids), ObPA-T (M1+Ob-PA+Tumoroids), Nw-MA-T (M0+Nw-MA+Tumoroids), and Ob-MA-T (M1+Ob-MA+Tumoroids) were normalized to their respective control (M0+Nw-PA, M1+Ob-PA, M0+Nw-MA, M1+Ob-MA) (red line) to follow the impact of DCIS-like tumoroids on each group. LIF, leukemia inhibitory factor; FAP, fibroblast activation protein; SMA-α, smooth muscle actin. **(D–F)** Impact of the Nw-TME and Ob-TME on macrophage polarization. THP-1 monocytes were activated into M0 and polarized into M1-like macrophages. **(D)** The quantification of each type of macrophages was measured by flow cytometry: Quantification of positive CD14+CD80- and CD80+ macrophage percentage is shown as bar graphs. Macrophages of adipose tissue co-cultured with adipocytes without cancer are used as control Nw-PA-M0 (M0+Nw-PA), Ob-PA-M1 (M1+Ob-PA), Nw-MA-M0 (M0+Nw-MA), Ob-MA (M1+Ob-MA).The gene expression of **(E)** proinflammatory (*IL-1β, IL-6, IL-8, TNFα*) and **(F)** anti-inflammatory (*TGF-β, IL-10, CD163*) cytokines was measured by qRT-PCR. β-actin was used as control. The four conditions Nw-PA-T (M0+Nw-PA+Tumoroids), Ob-PA-T (M1+Ob-PA+Tumoroids), Nw-MA-T (M0+Nw-MA+Tumoroids), and Ob-MA-T (M1+Ob-MA +Tumoroids) were normalized to their respective control (M0+Nw-PA, M1+Ob-PA, M0+Nw-MA, M1+Ob-MA) (red line) to follow the impact of DCIS-like tumoroids on each group. All data represent the means of 3-6 replicates ± SEM. *p<0.05, **p ≤ 0.01 ***p ≤ 0.001 represent significant differences compared with the control and # represents a significant difference between normal-weight and obese condition. nd, not determined.

DCIS-like tumoroids decreased the expression of genes involved in adipocytes (**Figure 3A**) through the downregulation of the expression of *PPAR-γ* and *AP2* (also known as FABP4), which are key regulators of adipogenesis and lipid metabolism.

In the presence of tumoroids, the expression of *AP2* was similarly downregulated by 50% in Nw-PA-T (*p ≤* 0.01), Ob-PA-T (*p ≤* 0.01), and Ob-MA-T (*p ≤* 0.001) while *PPAR-γ* was downregulated by 50% only within Nw-PA-T (*p ≤* 0.01) and Ob-PA-T (*p ≤* 0.01). This positive feedback loop between PPAR-γ and AP2 reinforces and maintains the pre-adipocyte phenotype. As a result, the adipocytes may maintain a more undifferentiated or less specialized state associated with decreased adiponectin levels.

Next, considering the importance of adipokines in obesity and related metabolic syndromes, the gene expression of *Adiponectin, HSL* (hormone-sensitive lipase), and *Leptin* was evaluated. The *Adiponectin* gene expression was significantly downregulated in all tumor microenvironments (TME), including Nw-MA-T (RQ=0.06, p ≤ 0.001), Ob-PA-T (RQ=0.03, p ≤ 0.001), and Ob-MA-T (RQ=0.14, p ≤ 0.001), and undetermined in Nw-PA-T. On the other hand, the gene expressions of *HSL* and *Leptin* were significantly increased, exhibiting the most potent effect within the two TMEs (Ob-PA-T and Ob-MA-T) associated with obesity.

##### 3.2.1.2 DCIS-like tumoroids may enhance CAF-like phenotypes and affect inflammatory cytokine expression

Following exposure to DCIS-like tumoroids, Nw adipose cells (PAs and MAs) might exhibit an iCAF phenotype with highly increased levels of *LIF* (Nw-PA-T: RQ=2.5, p ≤ 0.001; NwMA: RQ=1.8, p ≤ 0.05) (**Figure 3B**) and inflammatory cytokines (**Figure 3C**). However, following exposure to DCIS-like tumoroids, Ob-PA and Ob-MA expressed higher levels of all studied myoCAF genes (**Figure 3B**): *FAP* (Ob-PA-T: RQ=2.69, p ≤ 0.001; Ob-MA: RQ=1.7, p ≤ 0.05) and *α-SMA* (Ob-PA: RQ=2.54, p ≤ 0.001; Ob-MA: RQ=1.7, p ≤ 0.05). These iCAF-like phenotypes were positively correlated with a high increase in expression of *TNF-α, IL-1β, IL-8*, and *CXCL-10* whereas myoCAF-like phenotypes were positively correlated with a high increase in gene levels of *IL-6*, and high decrease in *TNF-α*. These changes lead adipocytes to produce pro-inflammatory cytokines. So, adipose cells associated with tumors from Nw individuals could adopt a cancer-educated inflammatory phenotype, whereas obesity mainly leads to the development of a myofibroblastic cancer-associated phenotype.

#### 3.2.2 DCIS-like tumoroids affected inflammatory cytokine expression and macrophage polarization

To determine the polarization state of macrophages (whether they were in an unpolarized M0 state or polarized into M1 or M2 phenotypes), we employed flow cytometry to analyze their specific CD markers. Then, we were able to quantitatively measure the percentage of each macrophage subtype (M0 and M1) relative to the control employing specific surface antibody labeling. The CD14^+^CD80^-^cell population can include both M0 and M2 macrophage subtypes. CD14 is a surface marker that is expressed on monocytes and macrophages (THP1/M0/M1/M2) and CD80 is expressed on pro-inflammatory M1-like macrophages. Similar to previous results, within the Nw-TME, CD14^+^CD80^-^ cell percentage significantly decreased to 64% in Nw-PA-T (p ≤ 0.01), while CD80^+^ M1 percentage increased to 20% in Nw-PA-T (p ≤ 0.001) and 13% in Nw-MA-T (p ≤ 0.05) comparing to control (NwPA+M0 -and Nw-MA+M0) (**Figure 3D**). The inflammatory state activated by adipocytes resembling to iCAF-like phenotype led to the polarization of macrophages into pro-inflammatory M1-like macrophages with high expression of pro-inflammatory cytokines (**Figure 3E**). However, DCIS-like tumoroids reduced the CD80^+^ M1 percentage within the obese TME: from 95% to 34.36% (Ob-PA-T, p ≤ 0.01) and from 89.9% to 39.7% (Ob-MA-T, p ≤ 0.01), while it increased the CD14^+^&CD80^-^ cell percentage from 9% to 20% (Ob-PA-T, p ≤ 0.001) and from 21.16% to 45% (Ob-MA-T, p ≤ 0.001), comparing to control (Ob-PA+M1, Ob-MA+M1) (**Figure 3F**). This increased percentage of CD14^+^CD80^-^ can be explained by the polarization of macrophages to the M2 phenotype that highly expressed the CD163 receptors may be due to the secretion of adipocytes having myoCAF-like phenotype and cancer promoter microenvironment.

### 3.3 Reciprocal interactions between adipose microenvironment and DCIS-like tumoroid through cytokine secretions

**Figure 4:**
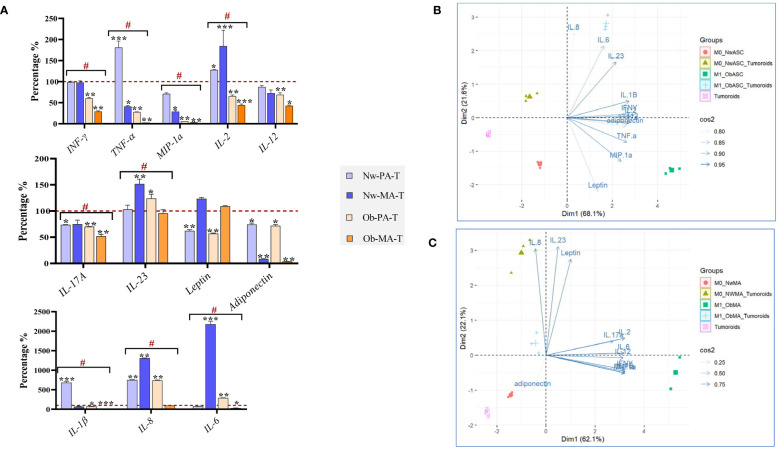
Evaluation of cytokine production in the 3D co-culture model. DCIS-like tumoroids were co-cultured with pre-adipospheroids (Nw-PA, Ob-PA) or adipospheroids (Nw-MA, Ob-MA) and with macrophages (M0 or M1). **(A)** Measurement of 12 pro-inflammatory cytokines levels (pg/μL) using Procarta-Plex™ Immunoassays was realized for each experimental condition. The percentages compared to the controls were calculated and presented as follows ± SEM. We used the Nw microenvironent (Nw-PA+M0) and the obese microenvironent (Ob-PA+M1) as controls for the normal weight and obese conditions respectively. Then we compared the variation between Nw and obese TME. All data represent the means of 3 replicates ± SEM *p < 0.05, **p ≤ 0.01, ***p ≤ 0.001 represent significant differences compared with the control and **#** represents a significant difference between normal-weight and obese condition. **(B, C)** Individual Principal Component Analysis (PCA) was realized to follow the 12 cytokine interactions within all Nw/Ob preadipose tissue **(B)** and Nw/Ob mature adipose tissue **(C)**. Cos2 (cosine squared) measures the quality of the representation of the variables on the principal components. **(B)** 75% of the variance of IL-8, IL-23, leptin, IL-17a, IL-2, IL-6, IL-12, IFN-γ, MIP-1α is explained by the selected principal component and 25% of the variance of adiponectin is explained by the selected principal component. **(C)** The selected principal component explains 95% of the variance of IL-23, IL-1, IFN-γ, adiponectin, TNF-α, and MIP-1α, 90% of the variance of IL-6, and 85% of the variance of leptin.

The reciprocal cell interactions in the co-culture 3D model were assessed on cytokine secretions by collecting conditioned media. As there was no contact between the different cell types in the experiment, the crosstalk was exclusively mediated by soluble factors. We used the conditions without tumoroids as controls and then we compared the variation between the normal weight and obese microenvironments (**Figure 4**).

#### 3.3.1 Pre-adipospheroids altered their cytokine secretions upon co-culture with DCIS-like tumoroids

Within both Nw-TME and Ob-TME, DCIS-like tumoroids significantly upregulated the production of IL-8, a pro-tumorigenic cytokine that stimulates migration and invasion to promote invasion and metastasis but downregulated the production of IL-17A, MIP-1α, leptin, and adiponectin (**Figure 4A**). In Nw-PA-T, tumoroids significantly increased the secretion of proinflammatory cytokines such as IL-1β, TNF-α, and IL-2 contrary to the Ob-PA-T. Ob-PA-T increased the production of IL-6 and IL-23 and decreased the production of certain pro-inflammatory cytokines such as TNF-α and IFN-γ, followed by the decrease of IL-2, IL-12, and IL-1β. There was no significant variation between Nw-PA-T and Ob-PA-T concerning the secretion of adiponectin, IL-17A, leptin, IL-8, IL-12, and IL-23, but there were significant variations in the other remaining cytokines between these 2 microenvironments. Therefore, we need to monitor more specifically the global interactions of these 12 cytokines as a function of the different microenvironments using PCA analysis (**Figure 4B**). In this analysis, we performed a two-dimensional PCA using appropriate statistical techniques and tools. We explored the relationship between the cytokines (89.7% of the total variable) and evaluated the ensemble (Dim1:68.1%; Dim2: 21.6%). Consistent with the previous results in **Figure 4A**, we found that inflammatory cytokines such as MIP-1α, TNF-α, IFN-γ, and leptin are the most dominant cytokines associated with inflammatory microenvironment with PA (Ob-PA+M1). Once this microenvironment was co-cultured with cancer, we found that the overall profile of these cytokines varied in the opposite direction to dimension 1 and approached the variables of dimension 2. This can be explained by the decrease in concentrations of inflammatory cytokines (Dim1, MIP-1α, TNF-α, IFN-γ, and leptin) and the increase in concentrations of IL-8, IL-6, and IL-23 (Dim 2).

#### 3.3.2 Adipospheroids altered their cytokine secretions upon co-culture with DCIS-like tumoroids

In both Nw-TME and Ob-TME, DCIS-like tumoroids significantly downregulated the production of adiponectin and MIP-1α, two anti-tumor cytokines, and decreased the secretion of pro-inflammatory cytokines such as TNF-α (**Figure 4A**). Within Nw-MA-T, tumoroids significantly decreased TNF-α production and increased the level of pro-inflammatory cytokines, notably IL-2, IL-23, IL-6, and IL-8. However, within Ob-MA-T, a strong reduction of pro-inflammatory cytokines such as TNF-α and IL-1β followed by decreases in IFN-γ, IL-2, IL-12, and IL-6 were noted. There was no significant variation between Nw-TME and Ob-TME regarding the levels of adiponectin, IL-17A, leptin, IL12, MIP-1α, and TNF-α, but there was significant variation in the rest of the cytokines between these 2 microenvironments. To link the global interactions of these 12 cytokines more specifically to the different microenvironments, a PCA analysis was carried out (**Figure 4C**) to explore the relationship between the cytokines (84.2% of total variable) and evaluate the whole (Dim1:62.1%; Dim2: 22.6%). Consistent with the previous results, we found that anti-inflammatory cytokines such as adiponectin were the most dominant cytokine in the Nw-TME (Nw-MA+M0). In addition, we noted that in the presence of cancer, the microenvironment (Nw-MA+M0+Tumoroids *vs* Nw-MA+M0) shifted in the opposite direction to dim 2 with significant decreases in adiponectin levels and increases in IL-8, IL-23, and leptin secretion.

## 4 Discussion

Using a 3D co-culture model between bi-fluorescent DCIS-like tumoroids (constituted by cancer and myoepithelial cells), adipose cells, and macrophages, the influence of the inflammatory adipose microenvironment found in the obese patient was investigated on DCIS progression. The reciprocal interactions between adipose tissue and DCIS were explored, including signaling pathways, phenotypic changes, and the effects of inflammation.

Our model revealed that obese conditions repress the expression of genes involved in apoptosis and promote genes involved in cell survival and inflammation. Indeed, Ob-PA-T and Ob-MA-T exhibited notable downregulation of apoptosis-related genes such as *BAX*, *BAG1*, and *CASP3*, surpassing the level observed in Nw-PA-T, with Nw-MA-T also showing decreased expression. A similar decrease in caspase gene expression (*CASP3, 8, 9*) was observed in both TMEs. This reduction reflects the suppression of TP53’s antitumor activity with an increase in the level of tumor propagation, which is followed by a reduction in the expression of the pro-apoptotic proteins BAX and BAG1, preventing apoptosis and promoting cell survival (40). This observation suggests an activation of the apoptotic signaling pathway, leading to caspase activation and the promotion of programmed cell death. Concurrently, it indicates the stimulation of tumor progression through the inhibition of MEC proliferation.

In addition, MMP-9 produced by tumor cells drives malignant progression and metastasis (41) and high levels of inflammatory cytokines such as TNF-α, IL-1β, Il-6, and IL-8 present in CM can also significantly increase MMP-9 expression (42, 43). TIMP1 (Tissue Inhibitor of Metalloproteinase 1) and MMPs are both involved in the regulation of extracellular matrix (ECM) remodeling, TIMP1 specifically inhibits the activity of several MMPs, including MMP-1 and MMP-9. The significant decrease in TIMP1 expression in Nw and Ob-TME cancer cells may contribute to tumor progression and elevated production of MMP9, an inducible enzyme that may also play an important role in angiogenesis (44).

In various glandular tissues, including mammary glands, ECMs act as a barrier and defense against cancer cell invasion (45). They play a role in inhibiting cancer cell invasion and migration, and in maintaining normal tissue architecture. In particular, α-SMA plays an important role in maintaining the structural integrity and contractile properties of various glandular tissues, including mammary glands. Loss of α-SMA expression in ECM from DCIS tumors has been associated with an increased risk of tumor progression and invasion (12). In addition, E-cadherin (CDH1), a cell adhesion molecule essential for epithelial cell integrity and tissue organization, is expressed in MECs and facilitates cell-cell adhesion via calcium-dependent interactions. The absence of E-cadherin expression in MECs cultured with all tumor microenvironment (TME) groups suggests a potential evolution towards a more migratory and invasive phenotype, disrupting the integrity of glandular tissue and allowing cancer cells to escape (46). Furthermore, the inflammatory and cancer-associated microenvironments induced in all studied groups may compromise ECM functionality, diminish their protective effects against cancer cells, and contribute to decreased viability and increased apoptotic pathways. These findings are consistent with previous research demonstrating a decreased MEC viability in inflammatory adipose microenvironments (11). Overall, our results suggest that obesity may promote the progression and invasiveness of ductal carcinoma *in situ* in the tumor microenvironment. Understanding these mechanisms could help identify factors contributing to DCIS development and facilitate the development of strategies to inhibit its invasion.

We showed that adipocytes significantly reduced their differentiation capacity under our experimental conditions, except Nw-MA-T, in agreement with a previous study reporting a reduced adipogenic differentiation capacity of ASCs from breast cancer TME (47). DCIS-like tumoroid can reduce adipocyte differentiation (PPAR-γ, AP2), decrease tumor-suppressor adipokine (adiponectin), and increase both adipocyte lipolysis (HSL) and tumor-enhancer adipokine (leptin) gene expression. Our results are consistent with a previous study that reported a similar reduction in the adipocyte differentiation capacity of ASCs in the context of invasive breast cancer (47). Overexpression of HSL, an enzyme (lipase E) involved in the degradation of stored triglycerides (lipolysis) in adipose tissue, in adipose cells (PA and MA) can indeed lead to increased lipolysis, which refers to the breakdown of stored lipids (fatty acids) in adipocytes. It has been suggested that tumor-induced factors or signals released by cancer cells may induce lipolysis in adipocytes (48, 49). This finding is in agreement with a previous study carried out on invasive ductal carcinoma (48, 49). Indeed, previous studies utilizing co-culture and 3D models have provided valuable insights into the metabolic interactions between adipocytes and cancer cells, specifically focusing on the transfer of lipids, particularly fatty acids (FFAs), from adipocytes to breast cancer cells (49). These studies have shown that under lean and obese conditions, breast cancer cells can efficiently capture FFAs released by adipocyte lipolysis by breaking down stored triglycerides. But this FFA transfer is enhanced under obese conditions, as obesity is associated with adipose tissue dysfunction, characterized by adipocyte hypertrophy, impaired adipokine secretion, and dysregulated lipid metabolism, which can increase FFA release and promote their uptake by cancer cells to provide an energy source supporting their growth and survival (50). The altered transcriptomic profiles observed in PAs within TME may indicate a potential dedifferentiation or reprogramming process, eventually leading to the development of a distinct subtype known as cancer-associated adipocytes (51). Finally, these findings underscored the critical role of cell interactions within TME highlighting how obesity-induced adipose tissue dysfunction can facilitate cancer progression.

On the other hand, tumor cells actively interact with adipose cells and manipulate them to create a supportive environment for tumor growth that may enhance CAF-like phenotypes and affect inflammatory cytokine expression (52). They may reprogram adipocyte phenotypes to cancer-associated fibroblast (CAF)-like and mesenchymal phenotype with proliferative capacity. CAFs are a heterogeneous population of fibroblasts that reside within the tumor stroma and play critical roles in tumor development and progression (53). Our findings revealed a strong correlation between iCAF-like phenotypes and increased expression of TNF-α, IL-1β, IL-8, and CXCL-10, while myoCAF-like phenotypes were associated with increased IL-6 levels and decreased TNF-α, suggesting distinct cytokine-driven pathways in adipocyte behavior (54). Particularly in Nw-PA-T conditions, adipocytes may adopt an iCAF-resembling phenotype, indicative of greater pro-inflammatory cytokine secretion, potentially contributing to an inflammatory microenvironment supporting cancer progression (55–57). Conversely, obesity-associated chronic inflammation likely promotes myoCAF-like phenotypes, emphasizing the significant impact of obesity on adipocyte dedifferentiation and tumor microenvironment dynamics (58). These results align with previous studies indicating a propensity for ASCs from obese patients to adopt myoCAF-like phenotypes upon exposure to low-malignant cancer cells (58). In addition, an ex-vivo correlation has been established, showing that breast cancer tissue sections from patients exhibit differential expression of the iCAF marker (LIF) (59–61), and the myoCAF marker (αSMA), depending on their obesity status (59, 62, 63). In Nw-MA, there may be a less pronounced but present iCAF phenotype, potentially correlating with elevated levels of immunomodulatory and inflammatory cytokines (57). Notably, tumoroids within the Nw-TME upregulate pro-inflammatory cytokines, suggesting a pro-inflammatory medium in this context. In contrast, adipocytes, macrophages, and cancer cells from the obese TME may exhibit dysfunctional behavior, possibly linked to myoCAF-like phenotypes (58). These myoCAFs can modulate immune cell activation and cytokine secretion, resulting in reduced pro-inflammatory cytokine levels and increased anti-inflammatory molecules such as IL-10, ultimately promoting an immunosuppressive environment (64). This dynamic interplay between obesity, inflammation, and tumor microenvironment highlights the multifaceted nature of adipocyte-macrophage interactions in cancer progression.

In obesity, macrophages tend to switch from an M2- to an M1-like phenotype, whereas in tumor progression, there’s often a switch from M1 to M2 macrophages (65), which creates an immunosuppressive microenvironment favoring tumor growth (66). CD163-positive macrophages, characteristic of the M2 alternatively activated macrophages, are associated with immunosuppression and tumor progression (67). Our findings corroborate existing literature, showing that TME conditions, particularly influenced by obesity, express high levels of immunosuppressive factors like TGF-β, IL-10, and CD163 (68) (69). Furthermore, our study highlighted the complex interplay between tumor cells and macrophages, as evidenced by the polarization of M0 macrophages towards an inflammatory M1-like phenotype in Nw-MA-T conditions, while Ob-TME promoted an anti-inflammatory response and polarization towards M2-like macrophages (70). This modulation of macrophage polarization within the TME reflects the dynamic nature of tumor-immune interactions and underscores the potential mechanisms driving tumor progression and immunosuppression.

Within both Nw-TME and Ob-TME, DCIS-like tumoroids upregulated IL-8 production, a pro-tumorigenic cytokine promoting migration and invasion (57, 71) while downregulating IL-17A, MIP-1α, leptin, and adiponectin. In Nw-PA-T, tumoroids notably increased the secretion of proinflammatory cytokines (IL-1β, TNF-α, and IL-2), capable of activating signaling pathways like STAT3, PI3K, and MAPK (72), and inducing EMT (73), unlike Ob-PA-T. This elevated proinflammatory cytokine secretion in Nw-PA-T, influenced by adipocytes resembling an iCAF-like phenotype and M1-like phenotypes, suggests a role in driving inflammation-associated tumor progression. Inflammatory cytokines TNF-α, IL-6, and IL-1β (57) may directly activate adipocyte signaling pathways, downregulating adiponectin and leptin production (74). Additionally, immunosuppressive factors from macrophages, like TGF-β and IL-10, inhibit IL-17A and MIP-1α production (69). IL-6 has been shown to trigger cancer cell proliferation by activating the JAK/STAT3, ERK1/2, and STAT3/NFĸB pathways in breast cancer cells and can lead to increased levels of IL-23, which is a link between tumor-associated inflammation and tumor immune evasion and can also activate NF-κB signaling (75). Our findings underscore the importance of considering the intricate balance between M1 and M2 macrophages in the TME and suggest potential therapeutic strategies targeting macrophage polarization to disrupt tumor-promoting immunosuppressive mechanisms.

In addition, various immunosuppressive factors produced by macrophages, such as TGF-β and IL-10, can inhibit IL-17A and MIP-1α production. Leptin, as an obesity-associated hormone present in co-cultured CMs, can reduce TNF-α levels through positive feedback (74).

Our findings strongly indicate that obesity significantly influences the tumor microenvironment, potentially impacting the trajectory of breast cancer development. These insights could hold great significance for guiding future targeted therapies aimed at addressing the complex interplay between obesity, inflammation, and tumor progression.

## 5 Conclusion

**Figure 5:**
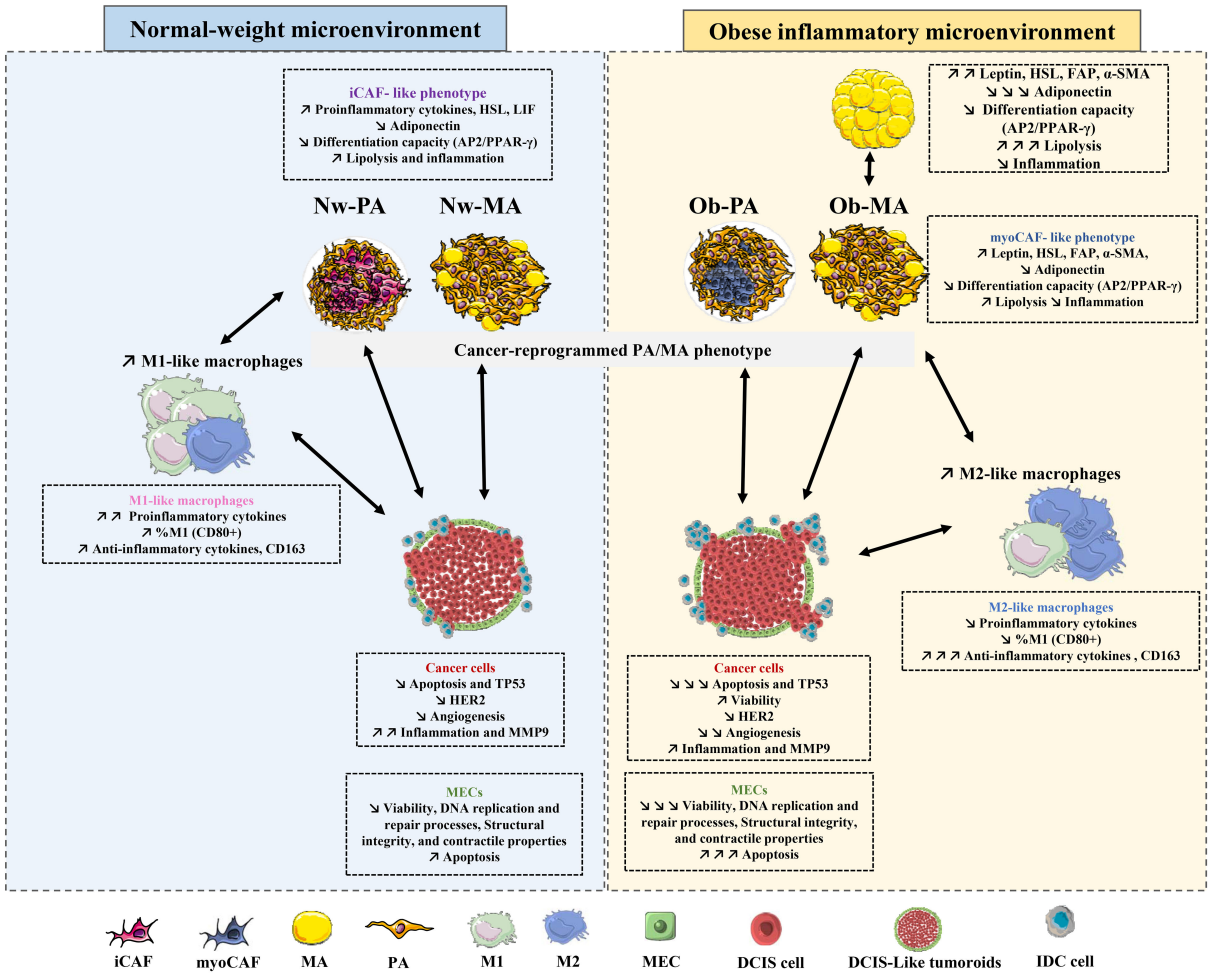
Graphical summary of the intercellular interactions between tumoroids, adipose cells, and macrophages.

In our present investigation, we have elucidated a substantial and reciprocal interplay between diverse constituents of the TME and DCIS-like tumoroids. Notably, the influence of TMEs derived from obese individuals exhibited a more pronounced impact compared to those from individuals with normal weight. Among the various TME components, Ob-PA-T demonstrated the most potent and predominant effect on reprogramming the entire TME, suggesting an increase in tumor progression and invasiveness. This intercellular communication can induce transcriptomic changes, reflecting alterations in gene expression patterns in adipose cells and macrophages, subsequently fueling the invasiveness of breast cancer cells and potentially disrupting the integrity of the MEC layer (**Figure 5**). Crucially, both Nw and Ob adipocytes changed their transcriptome and may adopt distinct iCAF or myoCAF-like profiles, respectively, thereby influencing macrophage polarization and repolarization. These adipose-tissue-associated alterations released a variety of factors, including inflammatory cytokines, adipokines, and extracellular matrix components, capable of promoting cancer cell invasiveness and proliferation. These factors activated signaling pathways in cancer cells, which can lead to increased migratory and invasive capacities. Furthermore, they stimulated cell cycle progression and supported cell survival, contributing to increased proliferation and decreased apoptosis of cancer cells. Moreover, the factors derived from adipose tissue could disrupt the integrity of the MEC layer surrounding DCIS cells, altering cell adhesion, polarity, and signaling pathways. This disturbance in the MEC layer compromises its tumor suppressor function, as the intact MEC layer serves as a physical barrier inhibiting cancer cell invasion and dissemination.

Although existing evidence suggests a connection between adipose tissue and its impact on cancer progression and the MEC layer, further studies are warranted to validate these findings and provide a more comprehensive understanding of the underlying molecular mechanisms.

## Data availability statement

The original contributions presented in the study are included in the article/**Supplementary Material**. Further inquiries can be directed to the corresponding author.

## Ethics statement

The surgical residue was harvested following French regulations including a declaration to the Research Ministry (DC no.2008162) and procurement of written informed consent from the patients.

## Author contributions

OH: Formal analysis, Investigation, Validation, Writing – original draft, Writing – review & editing. RN: Formal analysis, Investigation, Writing – review & editing. NG-M: Formal analysis, Methodology, Writing – review & editing. GC: Formal analysis, Methodology, Writing – review & editing. CB: Formal analysis, Methodology, Writing – review & editing. JA: Writing – review & editing, Formal analysis, Methodology. CD: Project administration, Writing – review & editing. CA: Project administration, Writing – review & editing. MD-A: Project administration, Writing – review & editing. FC-C: Conceptualization, Formal analysis, Funding acquisition, Investigation, Project administration, Validation, Writing – original draft, Writing – review & editing. LD: Conceptualization, Formal analysis, Funding acquisition, Investigation, Methodology, Project administration, Validation, Writing – original draft, Writing – review & editing.

## Glossary

**Table:**

ADF	adipocyte-derived fibroblast
AP2	adipocyte protein 2
ASC	adipose stem cell
BMI	body mass index
CAF	cancer-associated fibroblasts
CASP	caspase
CDH1	E-cadherin
CM	conditioned media
CXCL	C-X-C motif ligand
DCIS	ductal carcinoma *in situ*
EGFP	enhanced green fluorescent protein
EMT	epithelial-to-mesenchymal transition
ER	estrogen receptor
FAP	fibroblast activation protein
FFA	free fatty acid
HIF-1α	hypoxia-inducible factor-1α
GFP	green fluorescent protein
HR	hormone receptor
HSL	hormone-sensitive lipase
IDC	invasive ductal carcinoma
iCAF	inflammatory cancer-associated fibroblast
IFN-γ	interferon-γ
IL	interleukin
LIF	leukemia inhibitory factor
LPS	lipopolysaccharides
MA	mature adipocyte
MEC	myoepithelial cell
MMP	matrix metallopeptidase
MSC	mesenchymal stem cells
myoCAF	cancer-associated myofibroblasts
NF-κB	nuclear factor kappa-light-chain-enhancer of activated B cells
Nw-ASC	hASC from normal-weight women
PA	Pre-adipocyte
Nw-PA-T	normal-weight tumor microenvironment with Nw-PA
Nw-MA	MA from normal-weight women
Nw-MA-T	normal-weight tumor microenvironment with Nw-MA
Nw-TME	standard adipose tumor microenvironment found in normal-weight individuals
Ob-ASC	ASC from obese women
Ob-PA-T	obese inflammatory adipose tumor microenvironment with Ob-PA
Ob-MA	MA from obese women
Ob-MA-T	obese inflammatory adipose tumor microenvironment with Ob-MA
Ob-TME	inflammatory adipose tumor microenvironment found in obese people
PPAR-γ	peroxisome proliferator-activated receptor gamma
RFP	red fluorescent protein
SMA-α	smooth muscle actin
TAM	tumor-associated macrophage
TGF-β	transforming growth factor-β
TME	tumor microenvironment
TNFα	tumor necrosis factor-α
VEGF	vascular endothelial growth factor
