# Supplementary material for: The obese inflammatory microenvironment may promote breast DCIS progression
Source: Front Immunol. 2024 Jul 12;15:1384354. doi: 10.3389/fimmu.2024.1384354 (PMC11272476; doi:10.3389/fimmu.2024.1384354)
Supplement: Supplementary file 1 [file Presentation_1.pptx]

## Slide 1
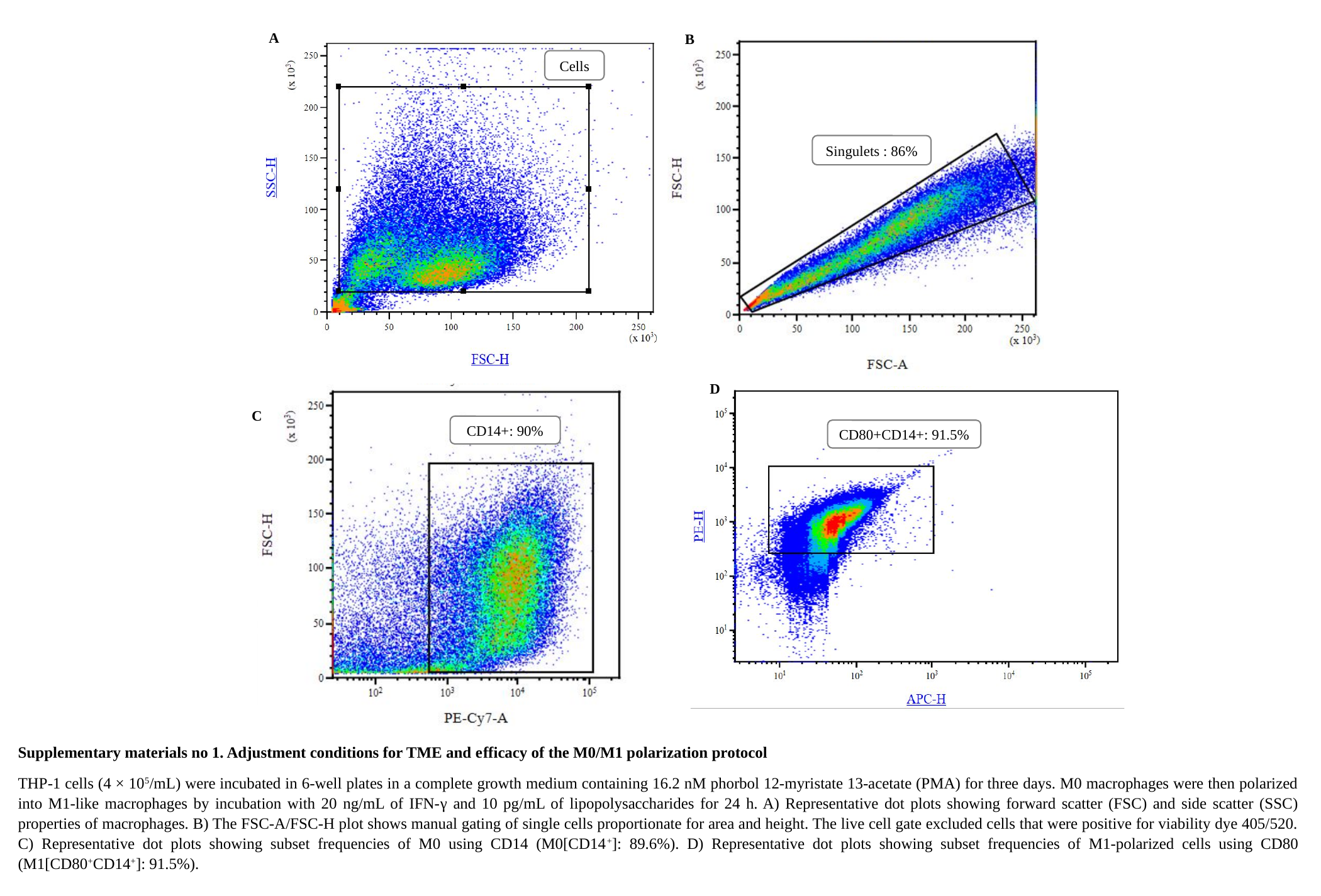

A
B
D
C
CD14+: 90%
CD80+CD14+: 91.5%
Cells
Singulets : 86%
Supplementary materials no 1. Adjustment conditions for TME and efficacy of the M0/M1 polarization protocol
THP-1 cells (4 × 10^5^/mL) were incubated in 6-well plates in a complete growth medium containing 16.2 nM phorbol 12-myristate 13-acetate (PMA) for three days. M0 macrophages were then polarized into M1-like macrophages by incubation with 20 ng/mL of IFN-γ and 10 pg/mL of lipopolysaccharides for 24 h. A) Representative dot plots showing forward scatter (FSC) and side scatter (SSC) properties of macrophages. B) The FSC-A/FSC-H plot shows manual gating of single cells proportionate for area and height. The live cell gate excluded cells that were positive for viability dye 405/520. C) Representative dot plots showing subset frequencies of M0 using CD14 (M0[CD14+]: 89.6%). D) Representative dot plots showing subset frequencies of M1-polarized cells using CD80 (M1[CD80+CD14+]: 91.5%).

## Slide 2
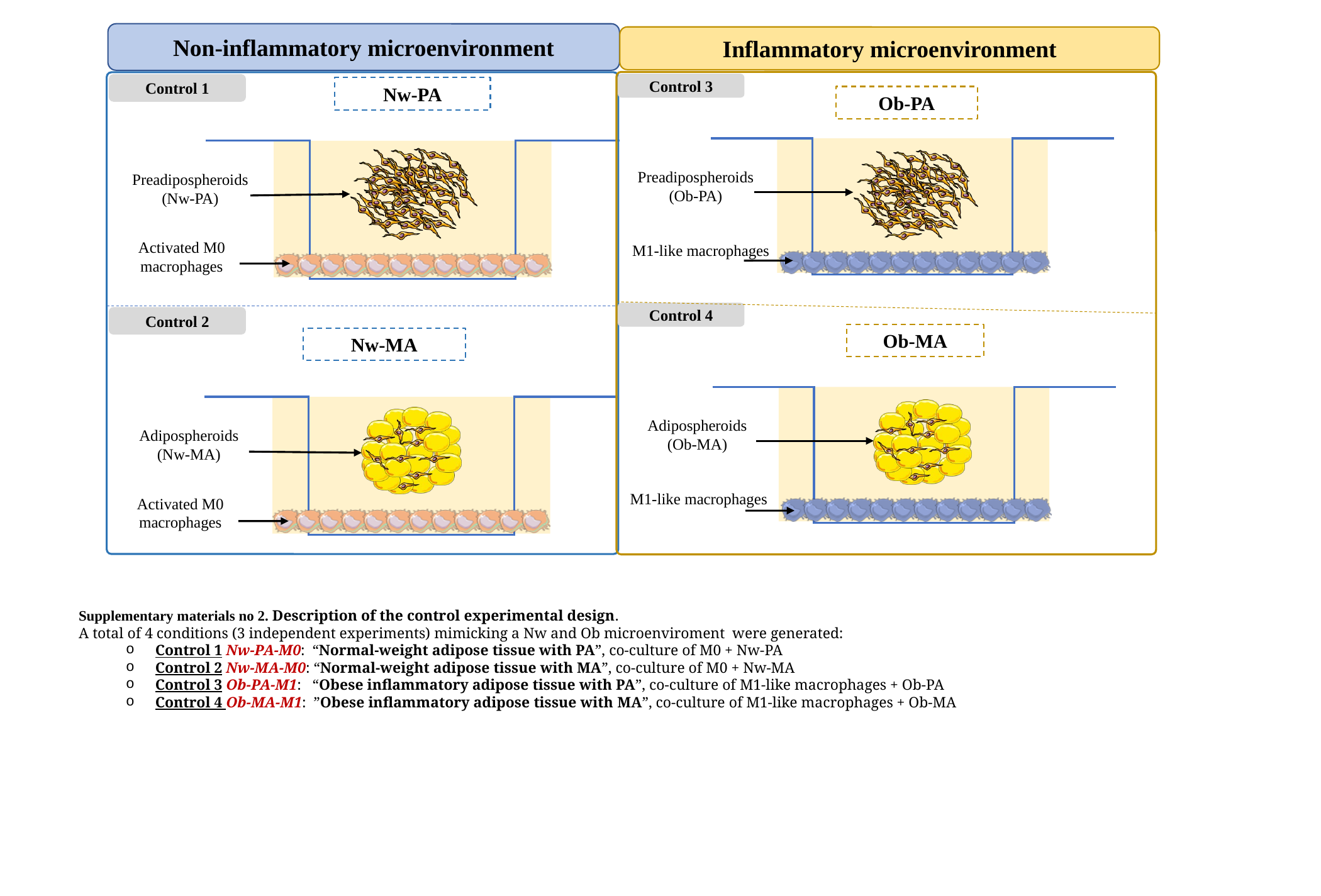

Non-inflammatory microenvironment
Inflammatory microenvironment
Control 3
Ob-PA
Preadipospheroids
(Ob-PA)
M1-like macrophages
Control 4
Ob-MA
Adipospheroids
(Ob-MA)
M1-like macrophages
Control 1
Nw-PA
Preadipospheroids
(Nw-PA)
Activated M0 macrophages
Control 2
Nw-MA
Adipospheroids
(Nw-MA)
Activated M0 macrophages
Supplementary materials no 2. Description of the control experimental design.
A total of 4 conditions (3 independent experiments) mimicking a Nw and Ob microenviroment were generated:
Control 1 Nw-PA-M0: “Normal-weight adipose tissue with PA”, co-culture of M0 + Nw-PA
Control 2 Nw-MA-M0: “Normal-weight adipose tissue with MA”, co-culture of M0 + Nw-MA
Control 3 Ob-PA-M1: “Obese inflammatory adipose tissue with PA”, co-culture of M1-like macrophages + Ob-PA
Control 4 Ob-MA-M1: ”Obese inflammatory adipose tissue with MA”, co-culture of M1-like macrophages + Ob-MA
